# Dissecting Phenotype from Genotype with Clinical Isolates of SARS-CoV-2 First Wave Variants

**DOI:** 10.3390/v15030611

**Published:** 2023-02-23

**Authors:** Mariah K. Taylor, Evan P. Williams, Yi Xue, Piroon Jenjaroenpun, Thidathip Wongsurawat, Amanda P. Smith, Amber M. Smith, Jyothi Parvathareddy, Ying Kong, Peter Vogel, Xueyuan Cao, Walter Reichard, Briana Spruill-Harrell, Amali E. Samarasinghe, Intawat Nookaew, Elizabeth A. Fitzpatrick, Micholas Dean Smith, Michelle Aranha, Jeremy C. Smith, Colleen B. Jonsson

**Affiliations:** 1 Department of Microbiology, Immunology and Biochemistry, The University of Tennessee Health Science Center, Memphis, TN 38163, USA; 2 Department of Biomedical Informatics, College of Medicine, University of Arkansas for Medical Sciences, Little Rock, AR 72205, USA; 3 Department of Pediatrics, The University of Tennessee Health Science Center, Memphis, TN 38103, USA; 4 Institute for the Study of Host-Pathogen Systems, University of Tennessee Health Science Center, Memphis, TN 38163, USA; 5 Regional Biocontainment Laboratory, The University of Tennessee Health Science Center, Memphis, TN 38163, USA; 6 Veterinary Pathology Core Laboratory, St Jude Children’s Research Hospital, Memphis, TN 38105, USA; 7 Department of Health Promotion and Disease Prevention, The University of Tennessee Health Science Center, Memphis, TN 38163, USA; 8 Center for Molecular Biophysics, University of Tennessee-Oak Ridge National Laboratory, Knoxville, TN 37996, USA; 9 Department of Biochemistry and Cellular and Molecular Biology, The University of Tennessee- Knoxville, Knoxville, TN 37996, USA

**Keywords:** clinical isolates, variants, pathology, immune response, replication, SARS-CoV-2, beta coronavirus, next-generation sequencing, single nucleotide polymorphism, K18-hACE2 transgenic mice

## Abstract

The emergence and availability of closely related clinical isolates of SARS-CoV-2 offers a unique opportunity to identify novel nonsynonymous mutations that may impact phenotype. Global sequencing efforts show that SARS-CoV-2 variants have emerged and then been replaced since the beginning of the pandemic, yet we have limited information regarding the breadth of variant-specific host responses. Using primary cell cultures and the K18-hACE2 mouse, we investigated the replication, innate immune response, and pathology of closely related, clinical variants circulating during the first wave of the pandemic. Mathematical modeling of the lung viral replication of four clinical isolates showed a dichotomy between two B.1. isolates with significantly faster and slower infected cell clearance rates, respectively. While isolates induced several common immune host responses to infection, one B.1 isolate was unique in the promotion of eosinophil-associated proteins IL-5 and CCL11. Moreover, its mortality rate was significantly slower. Lung microscopic histopathology suggested further phenotypic divergence among the five isolates showing three distinct sets of phenotypes: (i) consolidation, alveolar hemorrhage, and inflammation, (ii) interstitial inflammation/septal thickening and peribronchiolar/perivascular lymphoid cells, and (iii) consolidation, alveolar involvement, and endothelial hypertrophy/margination. Together these findings show divergence in the phenotypic outcomes of these clinical isolates and reveal the potential importance of nonsynonymous mutations in nsp2 and ORF8.

## 1. Introduction

Early in the coronavirus disease 2019 (COVID-19) pandemic, whole-genome sequencing (WGS) of nasopharyngeal (NP) swabs revealed that the introduction of severe acute respiratory syndrome coronavirus 2 (SARS-CoV-2) along the east and west coasts of the USA originated from travel to and from China and Europe [1,2]. Within the first month of the pandemic, SARS-CoV-2 diverged into two major lineages, A and B, and both entered the USA. A prominent mutation within the B lineage arose in early February 2020 in the spike (S) protein at D614G which gave rise to the B.1 lineage. This mutation was quickly demonstrated to confer greater stability in hACE2 receptor binding and, thereby, enhance the infectivity of cells expressing hACE2 [3,4]. Although betacoronaviruses (β-CoVs) have the largest genome among the known RNA viruses and have limited replication fidelity, no other dominant mutations emerged during the first wave of the pandemic with such an impact on phenotype. This can be explained by the relatively slow evolution of the virus following its introduction into human populations during the first wave [5]. In the search for signatures of selection in SARS-CoV-2 genomes from December 2019 through October 2020, no significant increases in selective pressures were discovered, although approximately 2% of the sites in the genes encoding the S and RNA-dependent RNA polymerase (RdRp) were under positive selection [5]. In contrast, a greater percentage of sites showed purifying selection, specifically 11.4% for the S and 7.2% for the RdRp. Hence, many of the mutations that have arisen have been transient, as they are removed via a purifying selection [6]. An outstanding question is whether any of these naturally occurring variants of SARS-CoV-2 that circulated during the first wave of the pandemic differed phenotypically.

Most of the in vitro and in vivo studies prior to the emergence of the variants of concern have focused on the SARS-CoV-2/USA-WA1/2020 isolate, hereby referred to as WA1/2020. The isolate came from the first confirmed case of COVID-19 in the USA, Washington, in January 2020, in a nonsmoking, healthy, 35-year-old man who had recently visited Wuhan, China [7]. The man was hospitalized but survived. WA1/2020 [8] has been evaluated in numerous studies and is widely used in the testing of vaccines, antivirals, and therapeutics in COVID-19 animal models [9,10,11]. The K18-hACE2 transgenic (K18-hACE2) mouse model has been instrumental in this testing and probing of the underlying mechanisms driving SARS-CoV-2 disease [10,12]. This mouse model shows robust replication of SARS-CoV-2 in the upper and lower respiratory tracts (URT and LRT) and severe interstitial pneumonia in the lung [12], resembling the clinical features of COVID-19. The model mirrors what is observed in persons with severe COVID-19, with the nasal cavity in the URT serving as the initial site for the entry, replication, and progression of infection and disease within the LRT [13,14,15,16]. Diffuse alveolar damage (DAD), inflammatory infiltrates, and foci of hemorrhage are common observations reported in microscopic examination of lungs during autopsy [17,18,19] and are prevalent in lung pathology in the COVID-19 K18-hACE2 mouse model. Lastly, the COVID-19 K18-hACE2 model demonstrates a dysregulated inflammatory response with robust IFN production [20,21] similar with findings in humans [22,23,24,25].

Given the diverse outcomes of COVID-19 disease and recovery in patients, we hypothesized that nonsynonymous mutations that have emerged in clinical isolates may confer additional phenotypes as compared to WA1/2020. Because the number of nonsynonymous mutations present within each clinical isolate was low during the first wave of the pandemic, we hypothesized that closely related viruses may enable the discovery of the phenotype caused by these mutations. To address this hypothesis, we conducted whole-genome sequencing (WGS) and the isolation of SARS-CoV-2 present in NP swab samples from deidentified qRT-PCR positive specimens collected during April 2020 from western Tennessee (TN), Alabama (AL), Arkansas (AR), and Mississippi (MS). Our in vitro and in vivo studies revealed notable and significant differences among four clinical isolates as compared to WA1/2020 in their replication, immune response, and/or pathology in mice. Of these, one isolate, UT29, was unique in the promotion of eosinophil-associated proteins IL-5 and CCL11 as well as in its significantly lower median survival.

## 2. Materials and Methods

### 2.1. Cell Culture

Chemicals and reagents were purchased from ThermoFisher unless indicated otherwise. Vero E6 cells (CRL-1586) were purchased from the American Type Culture Collection. SARS-CoV-2 viruses were grown in Vero E6 cells in complete minimal essential medium (MEM) with L-glutamine (Corning) containing certified 5% fetal bovine sera (FBS), 5 mM penicillin/streptomycin, and L-glutamine. Normal human tracheal epithelial cells (NHTE) donated from a 42-year-old Caucasian male were purchased through Cell Applications Cat# 504-05a, Lot# 2559. Normal human nasal epithelial cells (NHNE) cells collected from the nasal mucosa of a 48-year-old Caucasian male were purchased from PromoCell Cat# C12620, Lot# 464Z017.

### 2.2. SARS-CoV-2 Virus Isolates

SARS-CoV-2 human/USA/WA-CDC-WA1/2020 (WA1/2020) was obtained from BEI Resources Cat # NR-52281 and expanded to passage 3 (P3) in Vero E6 cells and sequenced to confirm no adaptive mutations were present in the spike. SARS-CoV-2 viral variants in this study included: SARS-CoV-2 human/USA/MS-UT5/2020 (UT5), human/USA/TN-UT12/2020 (UT12), human/USA/MS-UT21/2020 (UT21), human/USA/TN-UT23/2020 (UT23), human/USA/MS-UT27/2020 (UT27), and human/USA/MS-UT29/2020 (UT29), and they were isolated from nasopharyngeal (NP) swabs. All viral seed stocks were evaluated using next-generation sequencing as described in Section 2.4 and sequences were deposited at GenBank, accession no. OL365122-OL365125 and OL411643-OL411674. Briefly, 100 µL of each swab were used to infect Vero E6 cells in a 48-well plate for 30 min at 37 °C with 5% CO_2_ with occasional rocking. One mL of complete MEM was added to each well and the plate was monitored over five days for cytopathic effect (CPE). The supernatant was collected and clarified once the cells showed greater than 70% CPE (P1). The clarified virus was amplified further in a 6-well plate (P2) before expansion in a T175 (P3) and viral titer measured using a plaque assay (see Section 2.3). Virus seed stocks were frozen in 0.5 mL aliquots, kept at −80 °C until used and used only once. The consensus sequence of the swab was compared to the P3 seed stock to confirm no amino acid changes had occurred during expansion. All experiments using infectious viruses were conducted at BSL-3 or ABSL-3 areas of the UTHSC Regional Biocontainment Laboratory (RBL).

### 2.3. Plaque Assay

Virus titers were measured using a plaque assay of 10-fold serial dilutions using confluent Vero E6 cells in a 12-well plate. Briefly, after a 1 h incubation, the inoculum was removed, and each well was overlaid with a 1:1 solution of 2% CMC and 2× MEM and incubated for three days. After this time, the overlay was removed, cells were fixed with 10% formalin for 30 min, stained with 1% crystal violet solution for 10 min, and then washed twice with dH_2_O before reading.

### 2.4. Next-Generation Sequencing of NP Swabs and Virus Seed Stocks

A total of 200 µL of each NP swab or P3 virus seed stock was processed using the MagMAX Viral/Pathogen Nucleic Acid Isolation kit on the KingFisher Flex instrument following the manufacturer’s instructions (ThermoFisher, Waltham, MA, USA). RNA was eluted in 50 µL elution buffer and was processed for cDNA synthesis following ARTIC consortium guidelines with several exceptions. From each specimen, 11 µL of undiluted RNA were included in cDNA synthesis using SuperScript IV First Strand Synthesis System as well as 1 µL of a 60 µM random primer mix (NEB). The cDNA reaction was incubated in the thermocycler at 42 °C for 50 min, 70 °C for 10 min followed by a 4 °C hold. Two types of primers were used to produce amplicons, commercially available ARTIC version 3.0 (IdT) and a set of primers referred to herein as UT-V1 primers. Following PCR amplification (described below), purified PCR products were subject to library preparation using the Nextera XT DNA Library Preparation kit (Illumina) with corresponding Nextera XT Index Kit v2 (Illumina). The final library was measured on the Qubit Fluorometer 4 and loaded into the MiSeq Reagent kit v3 (150-cycle) (Illumina) and run on MiSeq instrument (Illumina).

### 2.5. Primer Design for UT-V1 Disjointed Tiling Amplicons

The design of the UT-V1 primers was based on an alignment of available SARS-CoV-2 genomes from the USA from GenBank using MUSCLE [26]. The alignment was uploaded into MEGA X [27], and used to create an ambiguous consensus sequence using the HIV Sequence Database [28] using the following parameters: Squeeze Gaps in Input = No, Consensus-by-Block Options = Default, Consensus Calculation Option = Default, Show both consensus + alignment = No. The consensus file was generated with wobbles and used to generate primers. Primers were designed such that each 500 nt region of the genome was covered twice, to produce 1 kb amplicons across the genome as well as 500 bp amplicons to cover the 5′ and 3′ ends of the genome. Primers were designed manually using the IDT OligoAnlyzer tool [29] and using the following parameters: oligonucleotide concentration 0.5 µM, Na+ concentration 0 mM, Mg++ concentration 1.5 mM, dNTP concentration 0.2 mM. Primers were designed with a primer length, GC content, and melting temperature of 18–26 nt, 40–60%, and 64–67 °C, respectively. NCBI Blast was also used to assess the E value of each primer using a coronavirus-specific search option, such that the ranges varied between 1 × 10^−5^ and 1 × 10^−8^ to ensure the specificity of each primer. Moreover, each primer was assessed using SARS-CoV-2 viral RNA purified from TRIzol LS reagent (Invitrogen). cDNA was synthesized using 10 µL RNA using the RevertAid First Strand cDNA Synthesis Kit (ThermoFisher) using the Oligo(dT)18 primer. Amplicons of 500 bp–1 kb amplicons were amplified using Phusion High-Fidelity PCR Master Mix with HF Buffer (ThermoFisher) with an annealing temperature of 60 °C and 1 µL cDNA and were individually assessed via gel electrophoresis.

### 2.6. PCR Amplification and Library Preparation

ARTIC primers were separated into two primer pools, and UT-V1 primers were separated into three primer pools. PCR reactions included 2.5 µL cDNA, 3.7 µL 10 µM primer pool, 6.3 µL nuclease-free water, and 12.5 µL Q5 Hot Start High-Fidelity 2× Master Mix (NEB). UT-V1 primer pools underwent PCR reactions as follows: denaturation at 98 °C for 30 s, followed by either 25 or 35 PCR cycles at 98 °C for 15 s, 60 °C for 5 min, and 63 °C for 30 s. ARTIC primers pools underwent denaturation at 98 °C for 30 s, followed by either 30 PCR cycles at 98 °C for 15 s and 63 °C for 5 min. PCR products were purified using AMPure XP beads (Beckman Coulter) and were washed with 80% ethanol. The concentration of dsDNA was measured using the Qubit dsDNA High Sensitivity kit. Library preparation and sequencing followed Oxford Nanopore Technology (ONT) PCR tiling of COVID-19 virus protocol version PTC_9096_v109_revE_06Feb2020.

### 2.7. Bioinformatics

Two methods were used to analyze sequences. For the first, the ONT raw reads (FAST5) were subjected to Guppy software version 3.4.5 for base-calling and demultiplexing. The ARTIC Network bioinformatics protocol [30] was used to generate consensus sequences for individual barcoded samples. Briefly, the demultiplexed FASTQ read files were passed through the quality control step by filtering the reads using the guppyplex script. The left-over reads were used in a reference-based assembly using the Medaka polishing software (ONT) against the reference sequence of the Wuhan-Hu-1 isolate (GenBank accession number MN908947.3). Single nucleotide variation calling was performed on the consensus sequences against the Wuhan-Hu-1 isolate as a reference using Snippy v4.4.0 [31,32].

For the second pipeline, FASTQ files of each library were imported into CLC Genomics Workbench v.21.0.3 (Qiagen). We generated the SNP data, QC for read mapping reports, QC for Sequencing Reads reports, and consensus sequences. This pipeline used a reference-based read mapping approach, where we used the WA1/2020 isolate (GenBank MN908947.3), which originated from an NP swab from a patient collected in January 2020, as a reference genome. We used two separate applications to evaluate SNPs. We compared this pipeline to Snippy v.4.4.0 [32].

High-quality consensus sequences were chosen within our dataset based on a QC < 20 as determined using NextClade v.0.13.0 [33,34]. These consensus sequences were assigned a lineage using the Pangolin lineage assessment epidemiological tool [35,36].

### 2.8. In Vitro Infection of Primary Epithelial Cells

NHTE cells at P4 and NHNE cells at P6 were seeded at 60,000 cells into 48-well cell culture plates. The next day, the P3 virus from WA1/2020, UT5, UT12, UT21, UT23, UT27, or UT29 was inoculated at MOI = 1. For 1 h, the plates were rocked every 15 min, washed twice, and then bronchial/tracheal epithelial cell growth medium (Cell Applications) or airway epithelial cell growth medium (PromoCell) was added. Cell culture supernatant was collected on 1, 2, and 3 dpi. To measure viral titers, a plaque assay was conducted from supernatant collected from NHNE or NHTE cells using Vero E6 cells as described above.

### 2.9. General Information and Parameters for Mouse-SARS-CoV-2 Studies

Male and female K18-hACE2 mice (B6.Cg-Tg(K18-ACE2)2Prlmn/J Mice, JAX: 034860) were purchased from Jackson Laboratories. Mice were identified with microchips (IPTT-300 by Bio Medic Data System) that were implanted subcutaneously. Seven-to-fourteen-week-old mice were used in these studies. The number of mice used in each study is provided in each figure legend. Following intranasal infection (15 µL per nare), the weight and temperature of all mice were taken daily, and mice were physically assessed and scored for signs of morbidity twice per day. All studies with SARS-CoV-2 were conducted in animal biosafety level 3 (ABSL-3). Studies were conducted in accordance with and approval of the Institutional Animal Care and Use Committee of University of Tennessee Health Science Center (Protocol #20-0132).

### 2.10. Assessment of WA1/2020 and Clinical Isolates in K18-hACE2 on 3 Days-Post-Infection

Male K18-hACE2 mice (12 weeks old) were placed under isoflurane anesthesia and then inoculated with either phosphate buffered saline (PBS) or with 1 × 10^4^ PFU of WA1/2020, UT5, UT12, UT21, UT23, UT27, or UT29). Animals were sacrificed at 3 dpi and the lung was separated into two halves. The left-half of the lung tissue was placed individually into bead mill tubes containing 1.4 mm ceramic beads, one mL PBS as well as 1× Halt Protease Inhibitor Cocktail. Tissues were homogenized using the Bead Mill 4 (Fisher Scientific) for three to four cycles of 10 s (five m/s) with 1 min intervals. Homogenized tissues were flash-frozen in liquid nitrogen and placed at −80 °C.

### 2.11. Survival Study of K18-hACE2 Mouse

Female K18-hACE2 mice were placed under isoflurane anesthesia and intranasally challenged with 1 × 10^4^ PFU of a third cell culture passage of either SARS-CoV-2 WA1/2020, or one of four clinical isolates (UT5, UT21, UT23, or UT29).

### 2.12. Assessment of WA1/2020 and Clinical Isolates in Male and Female K18-hACE2 Mice on 1, 3, and 5 Days Post-Infection

Male and female K18-hACE2 mice were placed under isoflurane anesthesia and then infected intranasally with PBS (mock) or 1 × 10^4^ PFU of SARS-CoV-2 WA1/2020, UT5, UT21, UT23, or UT29. Animals were sacrificed each on 1, 3, or 5 dpi, and the lung was separated into two halves. The left lobe of the lung tissue was placed into a Bead Mill tube (1.4 mm ceramic beads) containing 1 mL of PBS with Halt Protease Inhibitor Cocktail. The left lobe was homogenized using the Bead Mill 4 (Fisher) for 1–2 cycles of 10 s (5 m/s), centrifuged at 16,000× *g*, and the supernatant was aliquoted on ice and flash-frozen in liquid nitrogen before being placed in a −80 °C freezer. The remaining cell homogenate was suspended in 1 mL TRIzol Reagent and flash frozen. The right lobe of the lung was placed in 10% neutral buffered formalin and set aside in a 4 °C refrigerator until processed for histopathology or immunohistochemistry.

### 2.13. Multiplex Immunoassay

Cytokines in clarified homogenate from the left lobe of the lung were measured using a 36-multiplex immunoassay (ProcartaPlex Mouse Cytokine/Chemokine Convenience Panel 1A, ThermoFisher) following the manufacturer’s instructions using 25 µL as input material for each sample. The multiplex immunoassay was performed in the BSL-3 and the final 96-well plate run on the MAGPIX (Luminex), using Luminex XPONENT for MAGPIX software v.4.2. The data were normalized to levels in mock-infected mice and presented as a fold-change.

### 2.14. QuantiGene Plex Assay

To study gene expression profiles, total RNA from mouse lung homogenates of the left lobes were extracted using 500 µL TRIzol Reagent (Invitrogen). Total RNA was quantified using the Qubit RNA BR assay kit on the Qubit Fluorometer 4. We use 250 ng of RNA in duplicate to measure gene expression in a custom 50-plex mouse SARS-CoV-2 QuantiGene assay (ThermoFisher). The assay was performed in a BSL-3 facility, and the final plate was run on the MAGPIX and analyzed using Luminex XPONENT for MAGPIX software v.4.2.

### 2.15. Histopathology and Immunohistochemistry

Tissues were fixed in 10% neutral buffered formalin. Fixed lungs were then dehydrated, embedded in paraffin blocks, cut into 4 µm thick sections, and mounted on Superfrost Plus Microscope slides. Sections were then stained with hematoxylin and eosin and scored by an accredited veterinary pathologist (P.V.). For immunostaining of SARS-CoV-2 N protein, sections were deparaffinized and rehydrated in decreasing concentrations of ethanol. For antigen retrieval, a citrate-based (pH 6.0) solution was utilized to reveal SARS-CoV-2 N at 97 °C. Tissue sections were rinsed with deionized water, quenched with 1% hydrogen peroxide, washed with 1X PBS and incubated with primary rabbit antibodies against the SARS-CoV-2 N monoclonal antibody; 1:1000 (Sino Biologicals) were left on the slides to sit overnight at 4 °C. Slides were then washed three times in 1× PBS after which the biotinylated secondary anti-rabbit IgG antibody was applied at 1:200. Slides were rinsed three times with 1× PBS, stained with Vectastain Elite ABC-HRP kit (Vector Laboratories), washed three times with 1× PBS, and then the DAB solution was added. After rinsing, slides were counterstained with hematoxylin dehydrated in ethanol and xylene and mounted with a coverslip. Images were scanned in the Olympus SlideView VS200 (Olympus) using the Olympus OlyVIA v.3.2.1 imaging software.

### 2.16. Neutrophil Staining and Counting

Lung tissue sections were stained pink with Naphthol AS-D Chloroacetate Esterase (NACE), as previously described [37]. The slides were briefly deparaffined with xylene and rehydrated in decreasing concentrations of ethanol with deionized water. Slides were incubated for 30 min in NACE solution at 37 °C, counterstained with hematoxylin, and mounted with a coverslip using an aqueous mounting medium (Ted Pella).

In the Olympus OlyVIA program, whole tissue, bronchioles, respiratory ducts, lymphatic vessels, and blood vessels were outlined using the Freehand Polygon tool. To estimate the relative size of the whole tissue, the square area (m^2^) of the tissue was subtracted from the combined area of bronchioles, respiratory ducts, lymphatic vessels, and blood vessels. Neutrophils were identified across an entire tissue section and were counted by consideration of the color saturation, the number of lobes as evidenced by hematoxylin staining, clarity, and cellular size. To determine the density of neutrophils in the tissue, the number of NACE-positive cells was divided by the size of the tissue.

### 2.17. Quantification of Neutrophils via Flow Cytometry in SARS-CoV-2 Infection of K18-hACE2 Mice

Female K18-hACE2 mice were placed under isoflurane anesthesia and intranasally challenged with either a PBS (mock) or 1 × 10^4^ PFU of SARS-CoV-2 WA1/2020, UT5, UT21, UT23, or UT29. Mice were weighed daily and physically assessed for signs of morbidity. Animals were sacrificed on 5 dpi, and the whole lungs were weighed and enzymatically digested at 37 °C in 1 mg/mL collagenase (Sigma C0130), physically homogenized against a 40 µm cell strainer, and centrifuged at 675× *g* for 8 min. Supernatants were used to quantify viral loads using a plaque assay as described. Cell pellets were treated with red blood cell lysis buffer (Sigma R7767), washed with PBS followed by a FACS buffer (PBS, 5 mM EDTA, 10 mM HEPES, and 0.5% bovine serum albumin), counted with trypan blue exclusion using a Cell Countess System (Invitrogen), and prepared for flow cytometric analysis.

Single-cell suspensions were incubated with Fc receptor block (TruStainFcX, Biolegend, San Diego, CA) and viability dye (Zombie NIR Fixable Viability, Biolegend) (20 min, 4 °C) before surface staining (25 min, 4 °C) and fixation (25 min, 4 °C, BD Cytofix). Anti-mouse antibodies from Biolegend were used for cell subset analysis: Ly6G (clone 1A8, Brilliant Violet 421), F4/80 (clone BM8, Alexa Fluor 488), CD11c (clone N418, PE), CCR3 (clone J073E5, PE-Cy7), and Siglec-F (clone S17007L). Following fixation, samples were resuspended in FACS buffer and 50 µL of Count Bright Plus absolute counting beads (Invitrogen) were added to each sample. Samples were collected immediately (BD FACSAria; San Jose, CA, USA) and data were analyzed using FlowJo 10.7.2 (Tree Star, Ashland, OR, USA). Viable cells were gated from a forward scatter (FSC-A)/side scatter (SSC-A) plot, singlet inclusion, and viability dye exclusion. The high SSC-A cells were then gated into Ly6Ghi (neutrophils) and Ly6Glow/-subsets. Absolute count beads were gated from an FSC-A/SSC-A scatter plot followed by fluorescence in the PE channel. Absolute cells numbers (((total beads in sample)/(volume of sample (µL))) × (events in population gate)/(events in bead gate))) were scaled to the total number of cells isolated and normalized via tissue weight for statistical comparisons, and populations are plotted as the percent of viable cells per lung.

### 2.18. Statistical Analysis

Statistical analysis was performed using GraphPad Prism v.9.2.0 (GraphPad Software). Survival curves were compared using a log-rank (Mantel–Cox) test with the Bonferroni correction to establish a *p*-value significance of less than 0.005 between each group. For analyses of cytokine data, we used a 2-way ANOVA with Tukey’s multiple comparisons using a 95% confidence interval. Cells per mg of lung quantified via flow cytometry were compared using unpaired T tests with Welch’s correction. Protein and gene expression levels were log2 transformed to represent a normal distribution for the model. Statistical significance was considered if test differences contained a *p*-value less than 0.05.

Principal component analysis and its biplot were generated using the severity scores of seven pathological features (alveolar edema/hemorrhage, alveolar inflammation, interstitial inflammation/septal thickening, peribronchiolar/perivascular lymphoid cells, endothelial hypertrophy/margination, extent of alveolar involvement, and consolidation) using 1, 3, and 5 dpi and six isolates (mock, WA1/2020, UT5, UT21, UT23, and UT29). Standardized scaling was used to generate the PCA plot and its corresponding biplot. A scatterplot was created using principal component 1 (PC1) and PC2 values, and arrows were drawn in to connect averaged 1 and 5 dpi values.

## 3. Results

### 3.1. Subject Demographics, Next-Generation Sequencing, and Associated SARS-CoV-2 Lineages and SNPs

#### 3.1.1. Subject Demographics

NP swabs were selected from forty-five de-identified persons with a positive qRT-PCR test for SARS-CoV-2 in April 2020 for MinION sequencing from four states within continental USA. Of these, three (6.7%) were from AL, two (4.4%) were from AR, seveteen (37.8%) were from MS, and twenty-three (51.1%) were from TN. Persons were classified as either inpatient (*n* = 19, 42.2%) or outpatient (*n* = 26, 57.8%) at the time of the NP swab collection, and seventeen (37.8%) were male, twenty-five (55.6%) were female, and three (6.7%) individuals chose not to reveal their gender. Six (13.3%) subjects were <18 years, fourteen (31.1%) were aged 19–44 years, twenty-one (46.7%) were aged 45–64 years, three (6.7%) were aged 65–84 years, and one (2.2%) individual was >85 years. Due to the limitations of the approved Not Human Subjects Research (NHSR) IRB protocol, additional information on the samples was not available.

#### 3.1.2. Next-Generation Sequencing

**Figure 1:**
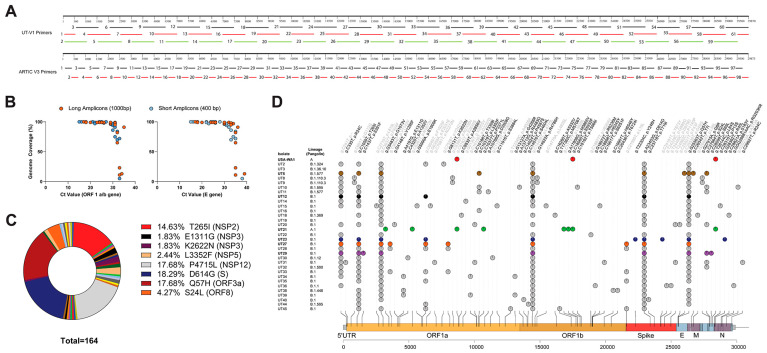
Single nucleotide polymorphisms and nonsynonymous mutations identified in SARS-CoV-2 nasopharyngeal samples. Schematic comparing two amplicon generation approaches (**A**) and the efficiency as defined by genome coverage as compared to Ct values (**B**). (**C**) Pie chart of the 162 nonsynonymous mutations identified from 31 sequences. The percentage of major nonsynonymous amino acid changes and corresponding gene location are listed to the right of the pie chart. (**D**) Variants associated with 31 high-quality clinical sample NGS sequences, and the SARS-CoV-2 WA1/2020 sequence, were mapped in comparison to the SARS-CoV-2 Wuhan-Hu-1 reference genome. Mutations observed across the genome are listed as gene (g.) or nucleotide change and its associated protein (p.) or amino acid change and variants are colored as synonymous mutations (grey) and nonsynonymous mutations (black). Mutations identified in virus isolates used in in vivo experimentation are colored as WA1/2020 (red), UT5 (brown), UT12 (black), UT21 (green), UT23 (navy blue), UT27 (orange), and UT29 (violet). Visualization of SNPs was designed using trackViewer v0.2.5 [39].

We used two primer schemes to generate amplicons for MinION library preparation, UT-V1, and Artic V3 (Figure 1A). We designed a UT-V1 disjointed primer amplicon tiling approach (i.e., adjacent primers are not in the same pool) that used sixty-one overlapping primer sets that were separated into three pools to generate 500 bp–1 kb amplicons (Figure 1A) [38]. The second approach used the ARTIC v3 primer set that generates ninety-eight 400 bp amplicons pooled into two PCR reactions. Both amplicon design schemes showed a genome coverage of 95–100% from NP swabs having Ct values < 30, and a genome coverage below 95% from NP swabs with Ct values > 30 (Figure 1B). The two primer sets showed drop-out regions at the ends of the genome, UT-V1 (approximately 1–460 nt and 29,580–29,903 nt), and ARTIC V3 (approximately 1–320 nt and 29,700–29,903 nt).

#### 3.1.3. Bioinformatics Reveal Six Variants with Unique Constellation of Nonsynonymous Mutations

Bioinformatic analyses revealed 31 high-quality sequences from the 45 NP samples (GenBank accession no. OL411643-OL411673) with NextClade [34] quality scores ranging from 0–18 (Figure 1D). Three sequences were obtained from samples from AL, one sample from AR, nine samples from MS, and nineteen from TN. The apparent cutoff of the Ct values required for ≥99% genome coverage was 30.94 (ORF 1ab gene) or 31.73 (E gene) (Figure 1B). Pangolin lineage analyses [36] of the consensus sequences for each of the thirty-one isolates suggested sixteen distinct lineages, twelve B.1., five B.1.411, and one A.1 lineage (Figure 1D). Interestingly, out of these 31 sequences, nonsynonymous mutations were not identified within the open reading frames of nsp6, nsp8, nsp9, nsp11, nsp16, ORF6, ORF7, or E (Table S3).

**Figure 2:**
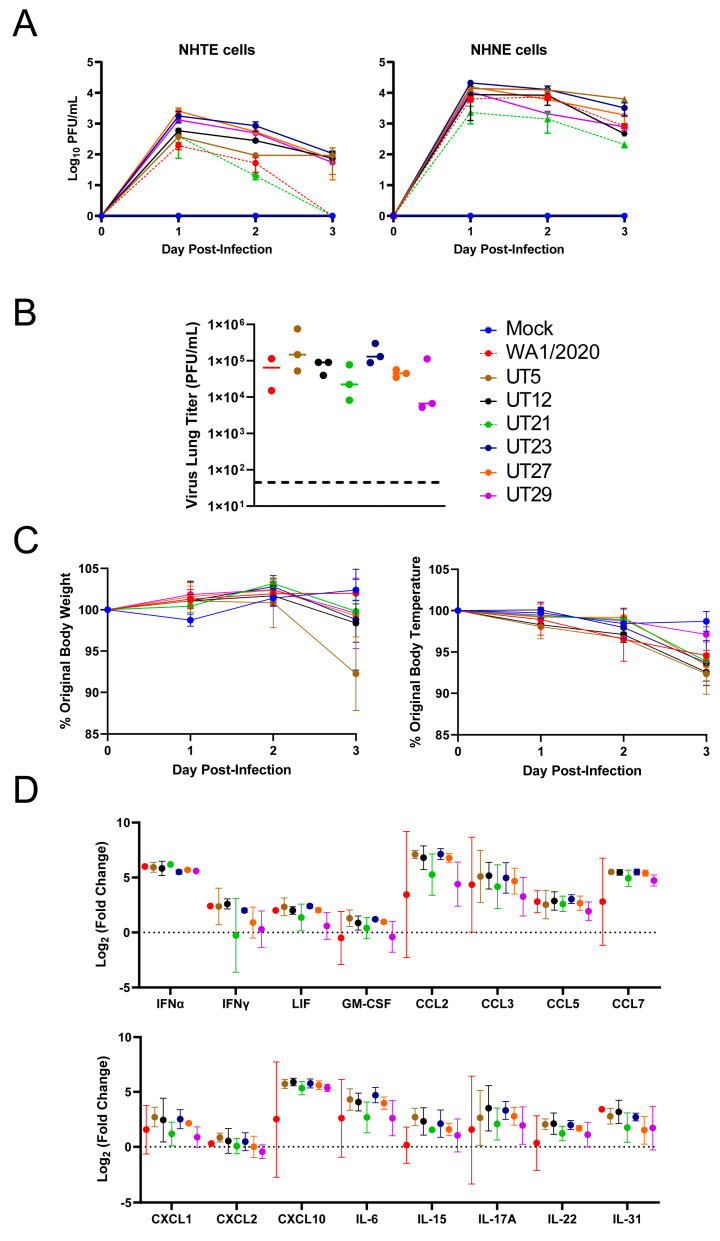
Evaluation of SARS-CoV-2 variants in primary epithelial cells and K18-hACE2 mouse model. (**A**) Normal human primary tracheal epithelial cells (NHTE) and normal human primary nasal epithelial cells (NHNE) were inoculated with a MOI = 1 and virus titer were assessed at 1 to 3 dpi with a plaque assay. Data are represented as mean ± standard error of the mean. Virus isolates within the A lineage, WA1/2020 (red), and A.1, UT21 (green), are represented by dashed lines. Virus isolates within the B.1 lineage, UT12 (black), UT23 (navy blue), UT27 (orange), and UT29 (violet), and B.1.577, UT5 (brown), are represented by solid lines. In B-D, female K18-hACE2 mice were intranasally challenged at 10^4^ PFU with either SARS-CoV-2 WA1/2020 (*n* = 2) or one of six clinical isolates UT5, UT12, UT21, UT23, UT27, or UT29 (*n* = 3). Graphs of lung viral titers (**B**), weight and body temperature (**C**), or selected immune responses in lung (**D**) are presented.

For experimental studies in primary cells and mice (Figure 2), we selected six isolates that had one to four unique nonsynonymous mutations excluding those sites conserved within A and B lineages (i.e., D614G) as compared to Wuhan-Hu-1 (Table 1); UT21 (A.1), UT5 (B.1.577), UT12, UT23, UT29 (B.1), and UT27 (B.1.411). Excluding lineage-defining mutations, each virus isolate contained one to four unique mutations. UT21 contained four unique nonsynonymous mutations, one in nsp3 (G1073V), one in nsp5 (P3371L), and two in nsp13 (P1427L and Y1464C). Disregarding the common lineage-defining mutations within lineage B and mutations observed in more than one isolate, UT5 had nonsynonymous mutations in nsp5 (L3352F) and M (T7I), UT23 had mutations in S (Y248H) and N (R40L), UT27 had a mutation in nsp3 (K2622N), and UT29 has mutations in nsp2 (S211F) and ORF8 (S69L). Evaluation of the prevalence of these mutations in the USA suggests many persisted and emerged in various lineages over time. The nsp2 S211F, for example, occurred in 99% of B.1.1.171 lineages and the P1427L circulated in 98% of B.1.1.288 lineages. The ORF8 S24L reached a prevalence of 20% across numerous lineages in more than 100 countries by the end of 2020 and then decreased until mid-2021 when it disappeared from circulation in B lineages. In contrast, ORF8:S69L continues to be present in SARS-CoV-2 genomes at less than 0.4% from the start of the pandemic and shows episodic increases in five different periods. The S211F, G1073V, P3371L, P1427L, Y1464C, Y248H, T7I, S69L, and R40L circulate below 0.5% in the USA and globally.

### 3.2. Replication Kinetics of Six Early SARS-CoV-2 Variants in Primary Epithelial Cells and Viral Load and Immune Responses in the Lungs of K18-hACE2 Mice

To evaluate potential phenotypic differences of the six genetically distinct clinical isolates (Table 1), we assessed replication in primary human cells (Figure 2A) and viral lung titer, weight, temperature, and host innate immune response at 3 days post-infection (dpi) in K18-hACE2 mice (Figure 2B–D). Approximately one log higher viral titers were noted for all isolates in NHNE cells as compared to NHTE cells at each time point (Figure 2A). UT21 had lower levels of replication than all other variants in NHNE cells. Replication of WA1/2020 and UT21 (lineage A isolates) in NHTE cells differed from lineage B isolates in that both were below the limit of detection at 3 dpi.

In K18-hACE2 mice infected with WA1/2020, UT5, UT12, UT21, UT23, UT27, or UT29, the viral load in the left lobe of the lung at 3 dpi reached 10^5^ plaque forming unit (PFU) for both UT5 and UT23, which was approximately one log higher than other isolates (Figure 2B). Mice infected with UT5 averaged an 8% drop in body weight compared to 0–2% for the other isolates (Figure 2C). Mice infected with UT29 showed the lowest average change (3%) in body temperature compared to the other six isolates (6–8%). Several cytokines including IFN-α, CCL2, CCL3, CCL4, CCL7, and CXCL10 were four to seven times higher at 3 dpi than mock-infected for all isolates (Figure 2D). Additionally, levels of CCL5, IL-6, IL-17A, and IL-31 were two to four times higher than the mock-infected group for all isolates (Figure 2D). Cumulatively, these data show a correlation between lung inflammation and viral load as is expected for viral pneumonia.

### 3.3. SARS-CoV-2 Clinical Isolates Yield Differences in Weight Loss and Viral Replication in the K18-hACE2 Mouse Model

Considering the phenotypic outcomes in vitro and in vivo (Figure 2), we down-selected four virus isolates, UT5 (B.1.577), UT21 (A.1), UT23 (B.1), and UT29 (B.1), and WA1/2020 was used as a comparator for additional in-depth studies to reveal any potential variant-specific host responses in the K18-hACE2 mouse model. We also considered the origin of the virus. UT5 and UT23 were from hospitalized patients, while UT21 and UT29 were from outpatient samples. The UT21 isolate was chosen for further studies as it was the only virus isolate in the A lineage.

**Figure 3:**
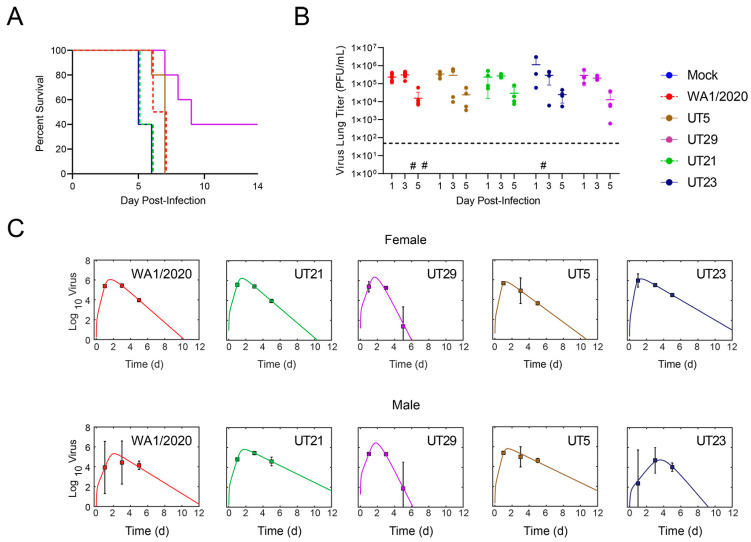
(**A**) Survival curves of female K18-hACE2 mice that were intranasally challenged at 1 × 10^4^ PFU with either SARS-CoV-2 WA1/2020 (*n* = 4) or one of four clinical isolates UT5, UT21, UT23, or UT29 (*n* = 5). The study was completed at 14 dpi. (**B**,**C**) K18-hACE2 mice (50:50 male: female) were intranasally challenged with either a mock, SARS-CoV-2 WA1/2020, or one of four clinical isolates at 1 × 10^4^ PFU, and were euthanized on 1, 3, or 5 dpi (*n* = 4 per timepoint). (**B**) The virus titer in the left lobe of the lung was measured via plaque assay (PFU/mL of half of the lung homogenate). The limit of detection of the plaque assay (horizontal dashed line) is 50 PFU. The symbol (#) represents one individual mouse without a measurable virus titer. (**C**) Fit of the viral kinetic model (Equations (S1)–(S4), Supplementary A) to viral loads from the lungs of female mice (top panel) and male mice (bottom panel) of WA1/2020 (red), UT21 (green), UT29 (violet), UT5 (brown), or UT23 (navy blue). Data are shown as geometric mean and standard deviation.

First, we measured the survival of female K18-hACE2 mice (*n* = 5/group) following intranasal infection with 10^4^ PFU of UT5, UT21, UT23, or UT29 (Figure 3A). The median survival time of mice infected with UT21 and UT23 was 5 days, WA1/2020 was 6.5 days, UT5 was 7 days, and UT29 was 9 days. The median survival of mice was significantly different between UT21/UT23 and UT29 (*p* = 0.003, Bonferroni correction *k* = 10).

In a separate cohort of mice (*n* = 3/virus), we evaluated the viral load in whole lung tissue (Figure 3B). Because our survival study showed that mice began to meet euthanasia criteria at 5 dpi, we chose 1, 3, and 5 dpi. Mice were infected intranasally with 1 × 10^4^ PFU and observed twice daily for clinical signs. Evaluation of viral titers from the left lobes of the lung showed a peak in titer at 3 dpi for all isolates (Figure 2C). UT23-infected mice had a slightly higher mean level of virus in the lung. To quantify the rates of growth and decay of virus load in the lung, we fit the model in Equations (S1)–(S4) (Supplementary A. Mathematical Modeling) to the viral loads from female mice infected with 1 × 10^4^ PFU WA1/2020, UT21, UT29, UT5, or UT23. The model yielded close fits to the data (Figure 2D) and resulted in the parameter estimates in Table S4.

Comparing the parameters (Table S4) suggested that UT29- and UT23-infected mice had significantly faster and slower infected cell clearance rates (δ), respectively, compared to the other isolates. In addition, the infectivity rates (β) of the UT5 and UT23 isolates were larger compared to WA1/2020, UT21, and UT29.

Fitting the model to the viral loads from male mice infected with the same dose and isolates again yielded a close fit to the data (Figure 2D) and suggested that UT23 had a rapid infected cell clearance rate (Table S4). In addition, UT29 also had rapid infected cell clearance compared to UT21 and UT5.

### 3.4. SARS-CoV-2 Variants Show Distinct Pathology Attributes in the K18-hACE2 Mouse Model

**Figure 4:**
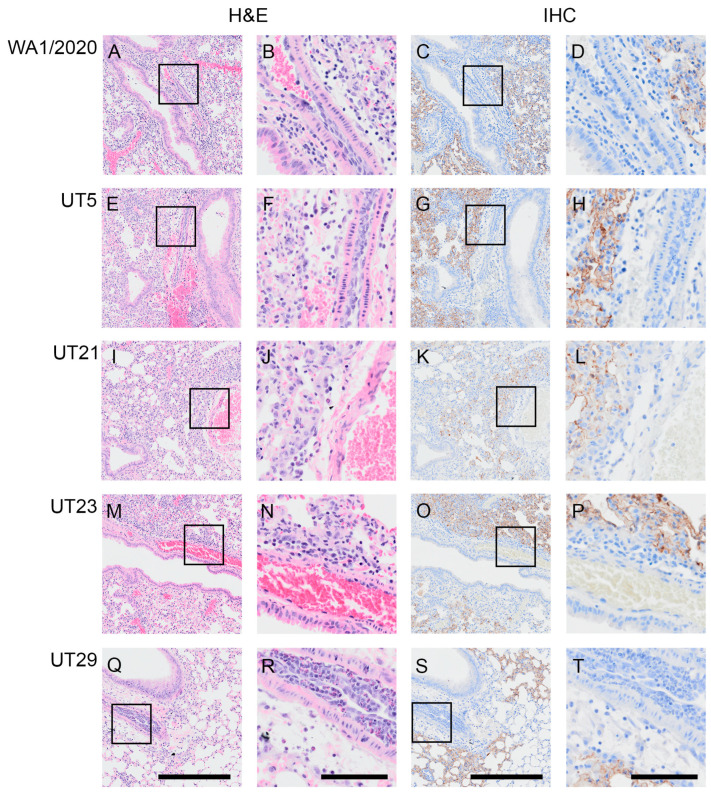
Margination and transmigration of inflammatory cells in pulmonary vessels of K18-hACE2 mice infected with WA1/2020, UT5, UT21, UT23, or UT29 at 5 DPI. Representative serial sections are presented for pulmonary vessels from mice at 5 dpi with WA1/2020 (**A**–**D**), UT5 (**E**–**H**), UT21 (**I**–**L**), UT23 (**M**–**P**), or UT29 (**Q**–**T**). Lungs were H&E-stained (**A**,**B**,**E**,**F**,**I**,**J**,**M**,**N**,**Q**,**R**) or IHC-labeled with antibody to the N protein of SARS-CoV-2 (**C**,**D**,**G**,**H**,**K**,**L**,**O**,**P**,**S**,**T**). The area outlined within each 100 µm image for each H&E and IHC set is shown to the right of each image. Scale bar = 100 µm for panels (**A**,**C**,**E**,**G**,**I**,**K**,**M**,**O**,**Q**,**S**). Scale bar = 25 µm for panels (**B**,**D**,**F**,**H**,**J**,**L**,**N**,**P**,**R**,**T**).

**Figure 5:**
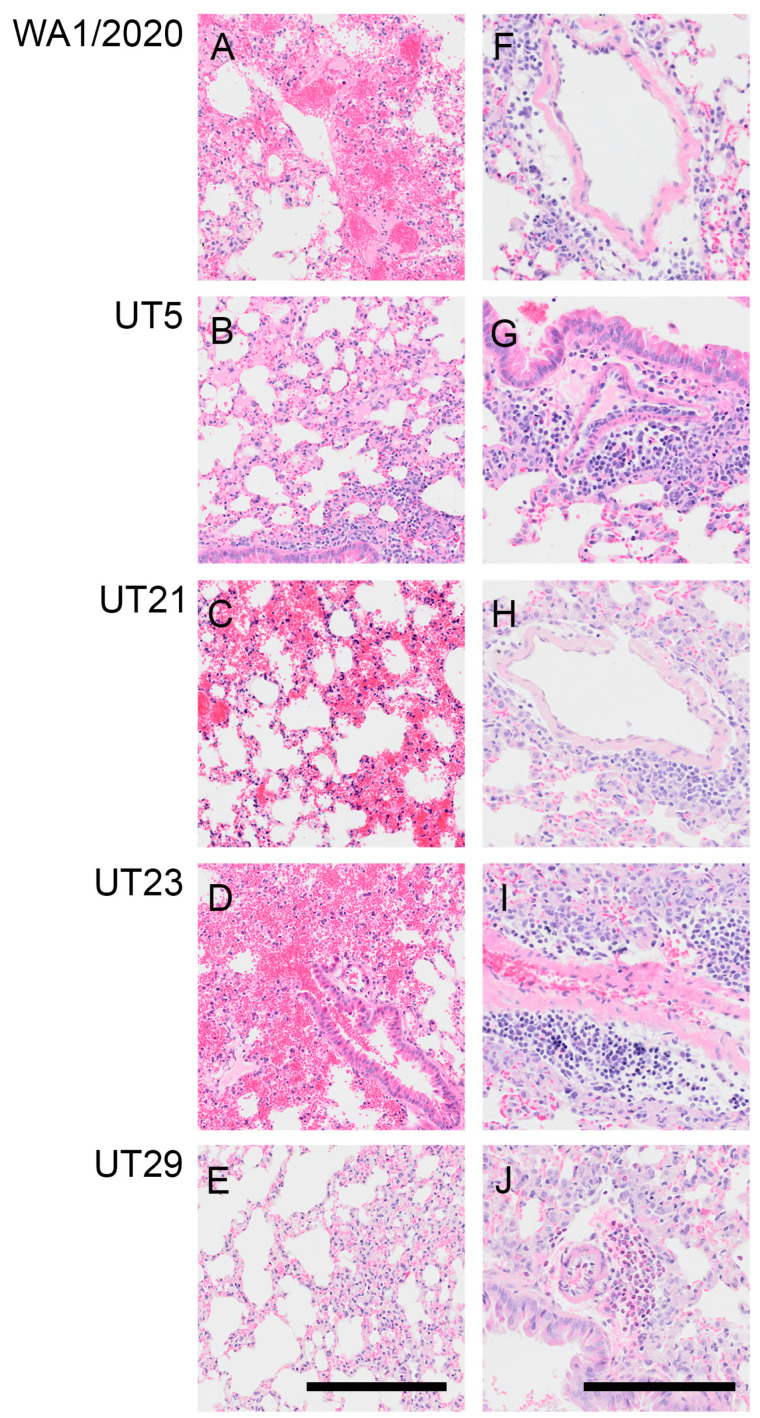
Representative images showing hemorrhage or perivascular inflammation in lungs of K18-hACE2 mice infected with WA1/2020, UT5, UT21, UT23, or UT29 at 5 DPI. Representative images of H&E-stained lung sections showing hemorrhage and necrosis from mice infected with WA1/2020 (**A**), UT21 (**C**), and UT23 (**D**). No hemorrhage was seen in UT5 (**B**) or UT29 (**E**). Lungs from UT5-infected mice showing vascular leakage (**B**) and monocyte infiltration (**G**). Representative images of lung sections with perivascular inflammation and edema characterized by infiltration of mononuclear cells from mice infected with WA1/2020 (**F**), UT5 (**G**), UT21 (**H**), UT23 (**I**), or tissue eosinophils in mice infected with UT29 (**J**). Edema is noted in (**F**,**I**). Scale bar = 25 µm.

In additional cohorts of male and female K18-hACE2 mice (n = two per sex per virus per timepoint), we scored microscopic lesions (Table S5), and evaluated lung pathogenesis (Figure 4 and Figure 5) and viral antigen distribution (Figure 4) on 1, 3, and 5 dpi. Minimal pathology was identified at 1 dpi across all infected mice, which was characterized by minimal focal infiltration of lymphoid cells in the perivascular sites. A marked increase in lesions was evident in H&E-stained SARS-CoV-2-infected lungs by 3 dpi, which demonstrated perivascular edema with mononuclear infiltrates, endothelial hypertrophy, interstitial pneumonia, and alveolar septal thickening. At 5 dpi, widespread pneumonia, as evidenced by perivascular edema and inflammation (mostly lymphocytic infiltrates), extensive consolidation, and thickened alveolar septa, was present in all infected mice (Figure 4). Additionally, margination and transmigration of inflammatory cells in pulmonary vessels at 5 dpi was prominent (Figure 4). The extent of alveolar involvement was greatest in WA1/2020-infected mice (Table S5). As demonstrated by immunohistochemical labeling of the N antigen, the virus was observed throughout lung tissue in all lobes by 3 dpi and increased in extent through 5 dpi (Figure 4).

At 5 dpi, lungs of mice infected with WA1/2020, UT5, or UT29 showed inflammatory cell accumulation in the alveolar spaces (compare Figure 5A,B,E). Mice infected with WA1/2020 or UT21 exhibited hemorrhaging, widespread edema necrotic debris in the lumen of the bronchus, and multifocal necrosis with inflammation (Figure 5A,E). At 5 dpi, hemorrhage was present in the lungs of UT5-, UT21-, and UT23-infected animals (compare Figure 5B–D), which was not observed during infection with UT29 (Figure 5E). Representative images are presented in Figure 5 and highlight perivascular inflammation and edema characterized by infiltration of mononuclear cells from mice infected with WA1/2020 (Figure 5F), UT5 (Figure 5G), UT21 (Figure 5H), and UT23 (Figure 5I). Clusters of abundant eosinophils were present throughout the vasculature in UT29-infected mice at 5 dpi (Figure 5E,J). No clusters of eosinophils were observed in our evaluation of H&E slides from lungs of all other variant-infected mice. Only a few dispersed eosinophils or no single eosinophils were viewed in WA1/2020, UT5, UT21, and UT23 lung sections (Figure 4 and Figure 5H–J).

**Figure 6:**
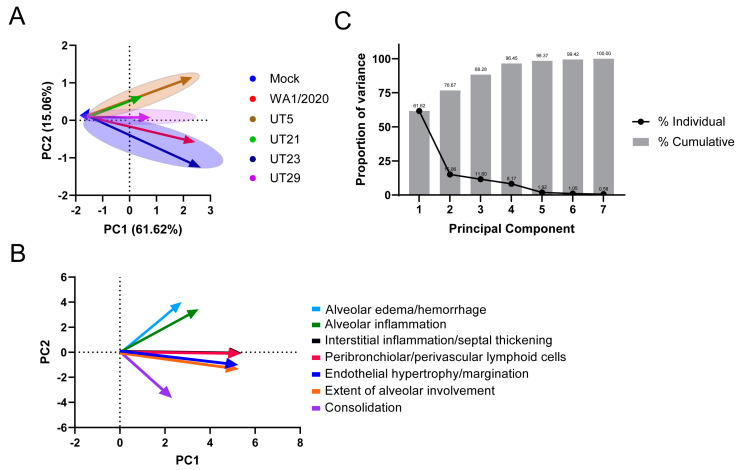
Principal component analysis of seven pathological features in lungs of K18-hACE2 mice infected with WA1/2020, UT5, UT21, UT23, or UT29. A principal component analysis (PCA) analysis of the pathology scores (Table S5) for mock, WA1/2020, UT5, UT21, UT23, and UT29 was conducted. Seven pathological features were scored (endothelial hypertrophy/margination, peribronchiolar/perivascular lymphoid cells, interstitial inflammation/septal thickening, alveolar inflammation, alveolar edema/hemorrhage, extent of alveolar involvement, and consolidation) for 1, 3, and 5 dpi. (**A**) The PCA plot distinguished the trend of each virus using 1 dpi (arrow tail) and 5 dpi (arrowhead) PC1 and PC2 values. (**B**) The PCA biplot is presented showing the association and trend of each parameter used for comparison, alveolar edema/hemorrhage (light blue), alveolar inflammation (green), interstitial inflammation/septal thickening (black), peribronchiolar/perivascular lymphoid cells (red), endothelial hypertrophy/margination (blue), the extent of alveolar involvement (orange), and consolidation (violet). (**C**) Proportion of variance from the principal component analysis of seven pathological features in lungs of K18-565 hACE2 mice infected with WA1/2020, UT5, UT21, UT23, or UT29. The line plot depicts the proportion of variance from each individual principal component (black dots), and the bar plot (grey bars) depicts the cumulative percentages of each of the principal components.

From seven microscopic pathological parameters used to score lung sections (Table S5), we performed a principal component analysis (PCA) using values from 1, 3, and 5 dpi to determine the clustering for each virus (Figure 6A,B). The seven parameters were the endothelial hypertrophy/margination, peribronchiolar/perivascular inflammatory cell infiltrates, interstitial inflammation/septal thickening, alveolar inflammation, alveolar edema/hemorrhage, and extent of alveolar involvement. We identified three PCA clusters based on the scoring for all isolates (Figure 6A). The WA1/2020- and UT23-infected cluster correlated with alveolar edema/hemorrhage and alveolar inflammation. UT29 formed its own cluster, which was associated with interstitial inflammation/septal thickening and peribronchiolar/perivascular lymphoid cells. The third cluster was UT5- and UT21-infected mice, which showed association with consolidation, alveolar involvement, and endothelial hypertrophy/margination.

**Figure 7:**
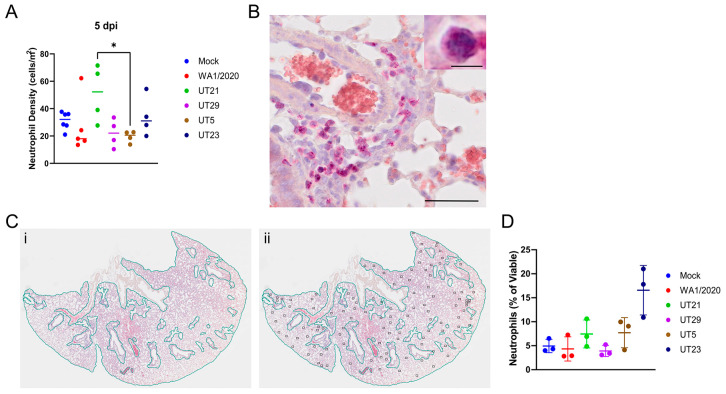
Quantification of neutrophil distribution and neutrophil density at 5 days pi within the lungs of SARS-CoV-2 infected mice. Neutrophils were stained pink with naphthol AS-D chloroacetate esterase (NACE) within lung tissues and counted. (**A**) Neutrophil density (cells/mm^2^) of each of the five SARS-CoV-2 virus isolates in *n* = 5 mice. * *p* < 0.05, one-way ANOVA with post-hoc Tukey HSD test. The adjusted *p*-value comparing UT5 and UT21 at 5 dpi is 0.0467. (**B**) Representative image to show NACE staining. (**C**) Method of calculating neutrophil density within lung tissue. (Panel (i)), the entire lung tissue was first outlined to obtain the area of the shape. Bronchioles, respiratory ducts, lymphatic vessels, and blood vessels were next outlined, and their combined square area was subtracted to estimate the total area of the tissue. (Panel (ii)), neutrophils were highlighted throughout the tissue and this number was divided by the total estimated area of the whole lung tissue. Only one lobe is shown for illustration of method. (**D**) The percentage of viable neutrophils at 5 dpi in the lungs of mice infected with five SARS-CoV-2 virus isolates in *n* = 3 mice. Graphs (**A**,**D**) show each virus with a distinct color and each point represents one mouse with the mean for each group.

To determine the extent of neutrophil infiltration, neutrophils within lung H&E tissue sections at 5 dpi were stained with NACE and counted (Figure 7A–C, Table S6). The number of neutrophils per area was calculated. No significant difference in neutrophil density was evident in one cross section of the whole lung between any virus isolate and the mock-infected mice, although animals infected with UT21 had a higher neutrophil density compared to those infected with UT5 (5 dpi).

To further quantify neutrophils levels, we used flow cytometry of whole lung digests at 5 dpi (Figure 7D). Neutrophils were significantly increased in UT23-infected animals compared to mice infected with any other isolate or mock-infected and the percent of neutrophils trended higher in UT21- and UT5-infected animals than in WA1/2020-, UT29-, and mock-infected controls.

### 3.5. Distinct Immune Response Profiles of SARS-CoV-2 Variants in K18-hACE2 Mouse Model

**Figure 8:**
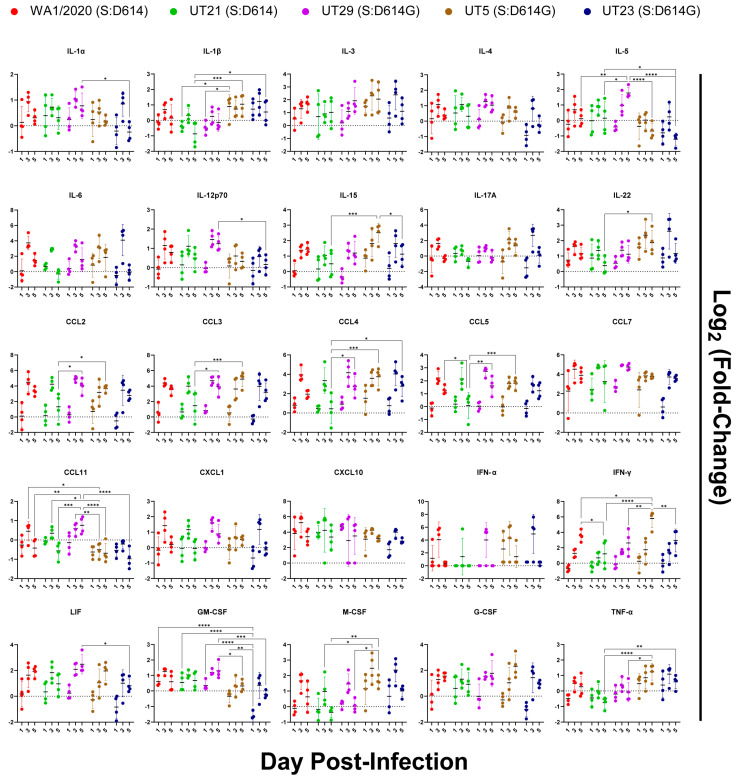
Immune protein response profiles of SARS-CoV-2 variants in lungs of K18-hACE2 mice (n = 4/virus per day) on days 1, 3, and 5 post-infection. Innate immune responses in lung following infection of K18-hACE2 mice with SARS-CoV-2 isolates were measured and protein concentration of each analyte were log2 (fold-change) transformed following comparison to mock. The mean and multiple comparisons at 95% confidence intervals are presented. Legend: WA1/2020 (red), UT21 (green), UT29 (violet), UT5 (brown), or UT23 (navy blue); * = *p* < 0.05, ** = *p* < 0.01, *** = *p* < 0.001, **** = *p* < 0.0001.

To evaluate the innate immune responses of the five SARS-CoV-2 variants, protein levels were measured in the left lobe of each lung using a 36-plex mouse panel (Figure 8 and Figure S3), and transcript levels were measured using a multiplexed gene expression panel (Table S7). Of the 36 cytokines tested, no significant changes were noted as compared to the mock-infected group for protein levels of IL-3, IL-4, IL-6, IL-17A, IL-27, CCL7, CXCL1, CXCL2, CXCL10, IFN-α, and G-CSF. Several cytokines did not show any significant difference across isolates (Figure 8). This included IL-3, IL-4, IL-6, IL-27, CXCL1, CXCL2, CXCL10, CCL7, GCSF, and IFN-α, most of which peaked at 3 dpi. Infection with all isolates resulted in an increase in IFN-γ from 1 to 5 dpi, and with UT5 having the highest level of expression. UT29-infected mice were unique in that they had significantly higher levels of IL-5 and CCL11 than most of the other isolates. Notably, on 5 dpi, UT29-infected mice resulted in greater levels of IL-5 and CCL11 in the lungs of mice compared to all other virus isolates. Additional differences were observed between the two isolates within lineage B.1, UT29 and UT23. UT29-infected mice had higher levels of CCL11, IL-1α, IL-12p70, LIF, and GM-CSF compared to UT23. IL-18 remained high in UT5 and WA1/2020-infected mice at 5 dpi (Figure S1). Compared to B lineage isolates, infection of mice with UT21 had lower levels of CCL2, CCL3, CCL4, TNF-α, and IL-1β.

**Figure 9:**
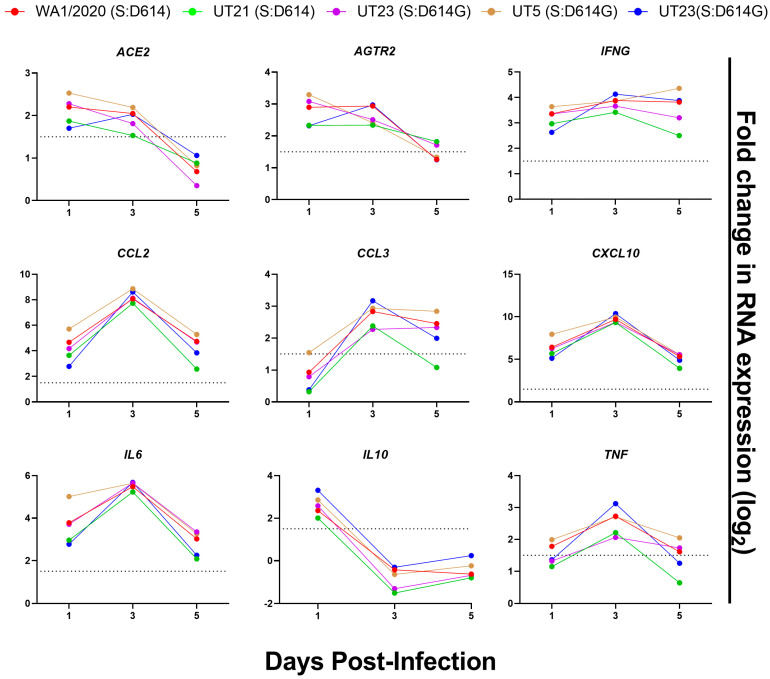
Selected gene expression profiles of lungs from K18-hACE2 inoculated with mock or SARS-CoV-2 variants. RNA was isolated from lungs (*n* = 4) harvested from mice on 1, 3, and 5 dpi and analyzed with a QuantiGene Plex panel targeting 50 genes (Table S7). V = To calculate gene expression, the measured average background fluorescent intensity of each measured gene was subtracted from mean fluorescent intensity of each gene in each sample. These values were normalized to that of an endogenous control within each sample. Normalized expression values were used to calculate log2 fold change as compared to that of mock-infected mice. Data are shown from WA1/2020 (red), UT21 (green), UT29 (violet), UT5 (brown), and UT23 (navy blue). The dotted line in each graph represents log2 fold change = 1.5.

Selected cytokines, chemokines, and other host responses were also evaluated in a multiplexed gene expression panel (Table S7, Figure 9). All virus isolates induced the mRNA expression of ACE2 and AGTR2, the receptor of ACE2, in the lungs upon infection, with the highest levels on 1 and 3 dpi followed by a drop to background levels by 5 dpi (Figure 9). As anticipated, there was no upregulation of DPP4 or TMPRSS2 (Table S7). Expression of CCL2, CCL3, CXCL10, TNF-a, and IL-6 mRNA peaked at 3 dpi and showed similar kinetics over time for all isolates. IL10 mRNA was highest at 1 dpi and dropped to below background levels on 3 and 5 dpi. Finally, the temporal dynamics of IFNG expression were similar for all variants except UT5, which continued to increase to 5 dpi.

## 4. Discussion

The rapid and continued emergence of new SARS-CoV-2 variants and expanding lineages have made the understanding of the phenotypic implications of specific amino acid mutations among clinical isolates challenging but immensely critical for understanding pathogenesis. In general, during the first year of the pandemic it was estimated that each lineage of SARS-CoV-2 accumulates two mutations each month [40]. The mutation rate is not across all proteins as different SARS-CoV-2 proteins and sites mutate at different rates [41]. The mutation rate is a result of the fidelity of replication and the proofreading mechanisms of the viral replication complex proteins, which have been estimated to have a mutation rate of 6.677 × 10^−4^ per site per year for the whole genome [42]. A common approach to defining the functional contributions of an amino acid is to engineer the change in a molecular clone. However, because the number of nonsynonymous mutations was minimal during the first wave, we recognized that closely related clinical isolates could serve as a bridge in the gap between genotype to phenotype. Hence the evaluation of closely related clinical isolates in primary cells and mouse models offers the advantage of quickly identifying novel nonsynonymous mutations that may impact phenotype.

Our findings regarding the replication of these viruses in primary cells and in mice suggested lineage-dependent differences in infectivity rates and immune-mediated infected cell clearance rates. We showed that the two A lineages, WA1/2020 and UT21, replicated poorly in NHTE cells as compared to NHNE cells, whereas the B lineages replicated well. This was not surprising because others have reported lower viral titers in primary cells infected with isolates that have the spike protein D614 compared to those with D614G, a mutation distinctly associated with the B lineage [43,44]. The D614G mutation has a greater binding affinity to the ACE2 receptor [45] and enhances the efficiency of entry [3]. One possibility for the slightly better replication of the A lineage in NHNE cells compared to NHTE cells may be due to the proportionally higher levels of the ACE2 receptor, AGTR2, and TMPRSS2 in these cells [46]. The replication dynamics in the mice were more complex, but the infectivity rates (β) were greater for UT5 and UT23 than WA1/2020 and UT21. In addition, UT29 had a rapid infected cell clearance rate as compared to UT21 and UT5, suggesting lineage-dependent differences in immune-mediated infected cell clearance rates.

Isolate-dependent differences were noted in the immune responses and lung pathology in infected mice. High levels of proinflammatory cytokines (i.e., IL-6) were present in the lung, which is similar to other reports for WA1/2020-infected K18-hACE2 mice [10,21]. We noted the peak fold change of proinflammatory and myeloid-associated cytokines at 3 dpi, and all isolates showed increasing production of IFN-γ through 5 dpi, although UT5 reached higher levels (Figure 8 and Figure 9). We noted higher IL-18 and TNF-α in UT5- and UT23-infected (B-lineage) mice as compared to UT21- (A lineage) and UT29-infected (B-lineage) mice (Figure 6 and Figure S3). In one study of COVID-19 cases, high serum IL-18 was associated with worse outcomes [47]. However, we found no significant correlation in our study with the elevation of any cytokine level, such as IL-18, or with worse outcomes in terms of mortality or pathology for any of these strains. Herein, UT5- and UT23-infected mice had more extensive alveolar edema and hemorrhage compared to mice infected with other isolates (Table S5). The most dramatic phenotypic distinction noted between UT29, and the other isolates were the distribution and proportion of eosinophils in the lungs (Figure 5). UT29-infected mice had distinct cytokine profiles compared to the other variant-infected mice, with higher levels of eosinophil-associated proteins IL-5, CCL5, and CCL11 (Figure 8). Additionally, UT29-infected mice had a median survival time of 9 dpi and had a 40% survival rate overall as compared to the 5- or 6-day median time of survival for all other isolates. The phenotypic differences induced by these variants in K18-hACE2 mice may be magnified in patients with different comorbidities and contribute to the heterogeneity in symptoms and disease severity observed in these groups.

SARS-CoV-2 A and B lineages were evaluated separately for amino acid changes that may have contributed to any differences in phenotype. Comparing the two A lineage isolates, UT21 and WA1/2020, there was no significant differences in the viral load or survival curves of mice, but there were higher pathological scores for WA1/2020 for consolidation and interstitial thickening. UT21 had higher scores for alveolar edema and hemorrhage. Additionally, WA1/2020-infected mice had significantly higher levels of IFN-γ, IL-18, and CCL5 at 5 dpi compared to U21-infected mice. UT21 has four nonsynonymous mutations as compared to WA1/2020 in nsp3 (papain-like protease, G1073V), nsp5 (P3371L, main protease), and nsp13 (RNA helicase, P1427L and Y1464C) (Table 1). The nsp3 G1073V is located within the macrodomain, which is a multifunctional region suggested to have involvement in viral pathogenicity [48], the disruption of host innate immunity [49], and is necessary for binding poly(ADP-ribose) polymerase 1 [50], which is involved in the regulation of cellular apoptosis. The two nsp13 mutations P1427L and Y1464C were noted in sequences obtained from passengers and crew sampled in early March 2020 aboard the Grand Princess cruise ship [51]. Introduction of P1427L and Y1464C causes a major structural alteration near the N-terminus, where a helix replaces a loop or chain-like structure, but no information is available as to how these two mutations might impact function [52]. Any one of these mutations in UT21 or all of them together may influence the dampened degree of innate immune signaling.

As previously mentioned, UT5 and UT23 were similar in their infectivity rates and pathology in mice. We observed a higher level of IL-18 and IFN-γ in the lungs of UT5-infected mice compared to UT23-infected mice on 5 dpi; however, there were no other notable differences between these two isolates in the immune response. UT5 has three nonsynonymous mutations, nsp5 (L3352F), M protein (T7I), and ORF8 (S24L), while UT23 has two mutations, one in the spike (Y248H) and one in the N (R40L) (Table 1). From early March to May 2020, S24L was a common mutation observed in ORF8 and was the second most prevalent mutation detected next to L84S [53]. During the time of this report, S24L was circulating at 13% prevalence in the United States. S24 may stabilize the dimeric interface within ORF8 [54]. Examination of the homology-based structural model of M [55,56], generated here using the I-TASSER package [57], indicates that the UT5-associated T7I mutation site exists within the protein’s short disordered ectodomain and is near a proposed glycosylation site located at N5 (Figure S2A). Interestingly, a previously reported study of murine hepatitis coronavirus indicated that a modulation of the ectodomain glycosylation of its M protein impacted host response [58]. Given the proximity of the M:T7I mutation to M:N5, glycosylation prediction calculations were performed (Supplementary A) and suggest that the mutant may inhibit the glycosylation of M:N5, and this in turn may contribute to differences in host immune response between UT5 and the other variants.

Mapping the non-conservative polar aromatic to basic mutation S:Y248H from UT23 onto the 3D structure of the protein indicates that it is located within the N-terminal domain (NTD) of the S1 subunit of the protein (Figure S2B) and is distal from the ACE2 RBD. In silico mutagenesis and short-molecular dynamics relaxations, along with informatics-based glycosylation analysis, indicate that the mutation induces no substantial changes in structure. The NTD has been reported to aid in the prefusion to post-fusion conformational change in the S protein [59], and the mutation is part of a functional binding interface (residues 246–260) of the NTD. Further, amino acid residue 248 is located in the N5 loop of the NTD which interacts with antibodies which have exhibited neutralizing activity to SARS-CoV-2, 4A8, COV2-2676, and COV2-2489 [60,61], and it is possible that the mutation from tyrosine to histidine abrogates the binding to functionally-important proteins and/or neutralizing antibodies that target the N-terminal domain and hence alter its pathogenicity. Lastly, the N:R40L mutation in UT23 has the potential to alter protein structure and solvent accessibility [53].

UT29 had the greatest phenotypic divergence from the other clinical isolates in replication kinetics, the median time to survival, pathology, and cytokine profile. In our histopathological evaluation of UT29-infected mice, we noted abundant eosinophil presence in the tissue and blood vessels of the lungs of male and female mice, which was supported by notable upregulation of eosinophil-associated cytokines at 3 and 5 dpi. COVID-19 disease severity in patients has been associated with eosinopenia, suggesting that increased eosinophils may lessen disease severity [62,63]. Early reports from COVID-19 patients showed that eosinopenia is associated with increased risk severity [62,64,65,66,67] and that the normalization of eosinophil counts was associated with hospital discharge [68]. As eosinophils do have antiviral properties against other respiratory viruses [69], it is possible that differences observed in eosinophil recruitment patterns by different strains investigated here may contribute to variations in disease severity observed in patients with high levels of lung eosinophils, such as asthmatics. Our future studies are aimed at investigating the role eosinophils play in SARS-CoV-2 infections.

UT29 has mutations in nsp2 (S211F) and ORF8 (S24L, S69L). Very little is known regarding SARS-CoV-1 or 2 nsp2 function beyond protein–protein interaction work that identified host interactions with several mitochondrial-associated proteins, prohibitin 1 and 2, and stomatin-like protein 2 [70]. ORF8 has been implicated in the downregulation of host innate immune pathways via the suppression of MHC-1 presentation [71] and immune evasion by suppressing type I IFN [72]. The S69L mutation is predicted to reduce the structural stability of ORF8 [73]. Patients infected with an ORF8:∆382 variant, which removes S24 and S69, had upregulated levels of proinflammatory cytokines but no demonstrable pneumonia [74]. Because UT5 also has the S24L mutation, one would not predict this mutation would contribute to the recruitment of eosinophils as clusters of eosinophils were not noted in the lungs of UT5-infected mice. The mutation at S211F in nsp2 may explain the high survival rates of UT29-infected mice. An analysis of the nsp2 cryo-EM/AI-derived structure (PDB: 7MSW) and N-terminal X-ray structure (PDB: 7EXM) [75] showed that S211 is located near one of three highly conserved zinc finger/ribbon motifs within 7 Å to 8 Å of the Zn within the CCHC (residues C190, C193, H202, and C236) zinc finger. Performing in silico mutagenesis using the MOE software package followed by a local energy minimization (using the default convergence criteria and the Amber14 force-field) [76] suggests that the S to F mutation, as a result of the substantially larger PHE sidechain, alters the structure of the CCHC zinc finger (RMSD 1.8 Å, Figure S3), including a substantial disruption of the zinc coordination. Given the strong evolutionary conservation of this zinc-binding motif, it is likely that the preservation of these domains is crucial for nsp2 function and thus, disruption of one of these fingers by the S211F mutation may deleteriously impact nsp2 function. Altogether, our results suggest that the nsp2:S211F in UT29, in combination with S69L, may have reduced disease severity in UT29-infected mice.

## 5. Conclusions

Similar to other areas in the USA, geographical hots spots of COVID-19 emerged in the southeast. At one point during the second wave in December 2020, Tennessee had the highest number of cases per 100,000 persons in the USA and continued to have the highest rates of infection in the USA during August–September 2021. While daunting, it is critical to understand the functional impact of mutations that emerged as well as those that continue to circulate and reemerge during the SARS-CoV-2 pandemic. Emphasis has been placed on mutations within the S protein, but we must also begin to unravel the functions of past and present mutations in the whole genome to appreciate a complete picture of the evolution of SARS-CoV-2 over the course of the pandemic. Evaluation of closely related clinical isolates in human primary cells and COVID-19 mouse models offers the advantage of quickly identifying novel mutations that impact phenotype.

## Figures and Tables

**Table 1:** Nonsynonymous amino acid mutations associated with six clinical isolates.

Protein	Nonsynonymous Mutation	WA1	UT21	UT12	UT23	UT29	UT27	UT5
nsp2	T85I							
	S211F							
nsp3	G255V							
	K1804N							
nsp5	L89F							
	P108L							
nsp12	P314L							
nsp13	P1427L							
	Y1464C							
S	Y248H							
	D614G							
ORF3a	Q57H							
M	T71							
ORF8	S24L							
	S69L							
	L84S							
N	R40L							

Cells in blue are those common to lineage A or B, while black denotes mutations in isolate.

## Data Availability

Sequences of the SARS-CoV-2 genomes generated for this manuscript have been deposited into GenBank and can be identified by GenBank accession no. OL365122-OL365125 and OL411643-OL411674.

## Supplementary Materials

The following supporting information can be downloaded at: https://www.mdpi.com/article/10.3390/v15030611/s1, Supplementary A and Supplementary B with Figure S1. Additional immune protein response profiles, Figure S2. Molecular renderings of M T7I and Spike protein Y248H mutants. Figure S3. Structural mapping of mutation on NSP2, Table S1. UT-V1 primers used for disjointed amplicon tiling approach, Table S2. MinION Sequencing Results, Table S3. Nonsynonymous amino acid changes for 31 SARS-CoV-2 consensus sequences, Table S4. Model Fit Parameters, Table S5. Pathology scoring of five SARS-CoV-2 isolates in K18-hACE2 mouse model, Table S6. Neutrophil counts at five dpi within sections of right lung lobes of infected mice, Table S7. Mouse Gene Expression Fold Change Results. Refs. [75,76,77,78,79,80,81,82,83,84,85,86,87,88,89,90,91,92,93,94,95,96] cited in Supplementary A.
