# Supplementary material for: Dissecting Phenotype from Genotype with Clinical Isolates of SARS-CoV-2 First Wave Variants
Source: Viruses. 2023 Feb 23;15(3):611. doi: 10.3390/v15030611 (PMC10059853; doi:10.3390/v15030611)
Supplement: Supplementary file 1 [file viruses-15-00611-s001.zip › Supplementary A/Supplementary A_Mathematical and structural modeling.pdf]

Mathematical modeling

We used a standard viral kinetic model in Equations S1-S4 which tracks susceptible epithelial ("target") cells (T), infected cells in the eclipse phase (I_1), infected cells producing virus (I_2), and free virus (V).

$$\frac{dT}{dt} = -\beta TV \qquad \text{(Equation S1)}$$

$$\frac{dI_1}{dt} = \beta TV - kI_1 \qquad \text{(Equation S2)}$$

$$\frac{dI_2}{dt} = kI_1 - \delta I_2 \qquad \text{(Equation S3)}$$

$$\frac{dV}{dt} = pI_2 - cV \qquad \text{(Equation S4)}$$

Here, target cells are infected with virus at rate βV per day. The cells then enter an eclipse phase (I_1) before transitioning at a rate k per day to cells that produce virus (I_2). Infected cells produce viruses at a rate *p* PFU/mL/cell/day. Free virus is cleared at a rate *c* per day, and infected cells are cleared at rate δ per day.

Given a parameter set θ, the cost C(θ) was minimized across parameter ranges using an Adaptive Simulated Annealing (ASA) global optimization algorithm [1] to compare experimental and predicted values of virus (V; $\log_{10}$ PFU/ml). Errors of the $\log_{10}$ data were assumed to be normally distributed. To explore and visualize the regions of parameters consistent with the model, we fit Equations 1-4 to 500 bootstrap replicates of the data. If the fit was within $\chi^2 = 0.05$ of the best-fit [1-3]. For each best-fit estimate, we provide 95% confidence intervals (CI) obtained

from the bootstrap replicates. Calculations were performed in MATLAB using a custom-built ASA algorithm [1], and *ode15s* as the ODE solver.

Estimated parameters included the rates of virus infection (β), virus production (*p*), and virus clearance (*c*). To reduce the parameter space due to limited data, the eclipse phase transition (k) was fixed to 4 per day, and the infected cell clearance (δ) was fixed to the slope of virus decay [4]. During bootstrap fits, we allowed the fixed value of the infected cell clearance rate (δ) to vary between ±20% to account for potential variability in this parameter. Bounds were placed on the parameters to constrain them to physically realistic values. The rates of infection (β) were allowed to vary between $1x10^{-6}$ and $5x10^{-3}$ $(PFU/ml)^{-1}$ d^{-1}, and the rates of virus production (*p*) between $1x10^{-2}$ and $5x10^{1}$ PFU/mL $cell^{-1}$ d^{-1}. Bounds for the virus clearance rate (*c*) were $1x10^{-2}$ and $5x10^{1}$ d^{-1}. The initial number of target cells (T(0)) was set to $1x10^{7}$ cells [1-3]. The initial number of infected cells $I_1(0)$ was set to $1x10^{4}$ cells to reflect an initial dose of $1x10^{4}$ PFU/ml [1]. We previously found that fixing or estimating $I_1(0)$ or fixing or estimating V(0) did not improve the fit and could not be statistically justified [1]. The initial number of productively infected cells ($I_2(0)$) and the initial free virus (V(0)) were set to 0.

Informatics-based analyses of UT5 M: T7I, UT23 S:Y248H mutations and nSP2:S211.
Complementary informatics-based estimates of fold-stability, coupled motions, and changes to glycosylation patterns were performed for a UT5 amino acid change on the M protein, T7I. Estimates of fold stability were made using SAAFEC-SEQ (http://compbio.clemson.edu/SAAFEC-SEQ/index.php) [5], MUpro (http://mupro.proteomics.ics.uci.edu/) [6], iStable (http://predictor.nchu.edu.tw/iStable/), [7], Molecular Operating Environment's (MOE) [8] residue scanning module, and I-Mutant2.0

(https://folding.biofold.org/i-mutant/i-mutant2.0.html) [9]. All web server-based estimates of stability made use of default parameters, while MOE-derived estimates made use of LowModeMD with the OPLS-AA force-field [10]. Estimates of coupled motions were determined using DynOmics web server (https://dyn.life.nthu.edu.tw/oENM/) [11] using the MembraneANM options and input prepared with the positioning of proteins in membrane web server (https://opm.phar.umich.edu/ppm_server2) [12]. Prediction of N- and O-linked glycosylation sites were performed on the NetNGlyc 1.0 server (http://www.cbs.dtu.dk/services/NetNGlyc/) [13] and SPRINT-Gly (https://sparks-lab.org/server/sprint-gly/) [14]. This M: T71 mutation site was mapped onto a recently developed homology model [15] and generated with the I-TASSER homology package [16].

For the spike protein, we used the GROMACS simulation package [17] in conjunction with default simulation parameters, i.e. non-bonded cutoffs of 1.2 nm along with PME electrostatics and Nose-Hoover/Parinello-Rahman thermo/barostats, taken from CHARMM-GUI [18, 19], a set of short (2ns) molecular dynamics simulations of the mutant and wild type spike, with the initial structures taken from a structural alignment, and *in silico* mutagenesis and glycosylation performed using the Molecular Operating Environment software package [8] of the PDB entries 6M0J [20] and 6VXX [21].

An analysis of the nsp2 cryo-EM/AI derived structure (PDB: 7MSW)[22] and N-terminal x-ray structure (PDB: 7EXM)[23] showed that S211 is located near one of three highly conserved zinc-finger/ribbon motifs[22]. S211 is within 7Å to 8Å of the Zn within the CCHC (residues C190, C193, H202, and C236) zinc finger. Performing *in silico* mutagenesis using the MOE software package followed by local energy minimization (using the default convergence criteria and the

Amber14 force-field)[24], suggests that the S to F mutation, as a result of the substantially larger PHE sidechain, alters the structure of the CCHC zinc-finger (RMSD 1.8Å, figure S3), including a substantial disruption of the zinc coordination. Given the strong evolutionary conservation of this zinc-binding motif it is likely that the preservation of these domains are crucial for nsp2 function and thus disruption of one of these fingers by the S211F mutation may deleteriously impact nsp2 function.

References

1. Smith, A. P.; Moquin, D. J.; Bernhauerova, V.; Smith, A. M., Influenza Virus Infection Model With Density Dependence Supports Biphasic Viral Decay. *Front Microbiol* **2018,** 9, (1554).
2. Smith, A. M.; Adler, F. R.; McAuley, J. L.; Gutenkunst, R. N.; Ribeiro, R. M.; McCullers, J. A.; Perelson, A. S., Effect of 1918 PB1-F2 Expression on Influenza A Virus Infection Kinetics. *PLOS Computational Biology* **2011,** 7, (2), e1001081.
3. Smith, A. M.; Adler, F. R.; Ribeiro, R. M.; Gutenkunst, R. N.; McAuley, J. L.; McCullers, J. A.; Perelson, A. S., Kinetics of Coinfection with Influenza A Virus and Streptococcus pneumoniae. *PLoS Pathog* **2013,** 9, (3), e1003238.
4. Smith, A. M.; Adler, F. R.; Perelson, A. S., An accurate two-phase approximate solution to an acute viral infection model. *J Math Biol* **2010,** 60, (5), 711-26.
5. Li, G.; Panday, S. K.; Alexov, E., SAAFEC-SEQ: A Sequence-Based Method for Predicting the Effect of Single Point Mutations on Protein Thermodynamic Stability. *Int J Mol Sci* **2021,** 22, (2), 606.
6. Cheng, J.; Randall, A.; Baldi, P., Prediction of protein stability changes for single-site mutations using support vector machines. *Proteins: Structure, Function, and Bioinformatics* **2006,** 62, (4), 1125-1132.
7. Chen, C.-W.; Lin, J.; Chu, Y.-W., iStable: off-the-shelf predictor integration for predicting protein stability changes. *BMC Bioinformatics* **2013,** 14 Suppl 2, (Suppl 2), S5-S5.
8. MolecularOperatingEnvironment 2019.01; Chemical Computing Group ULC 1010 Sherbooke St. West, Suite #910, Montreal, QC, Canada, 2022.
9. Capriotti, E.; Fariselli, P.; Casadio, R., I-Mutant2.0: predicting stability changes upon mutation from the protein sequence or structure. *Nucleic acids research* **2005,** 33, (Web Server issue), W306-W310.
10. Kaminski, G. A.; Friesner, R. A.; Tirado-Rives, J.; Jorgensen, W. L., Evaluation and Reparametrization of the OPLS-AA Force Field for Proteins via Comparison with Accurate Quantum Chemical Calculations on Peptides. *The Journal of Physical Chemistry B* **2001,** 105, (28), 6474-6487.
11. Li, H.; Chang, Y. Y.; Lee, J. Y.; Bahar, I.; Yang, L. W., DynOmics: dynamics of structural proteome and beyond. *Nucleic Acids Res* **2017,** 45, (W1), W374-w380.

12. Lomize, M. A.; Pogozheva, I. D.; Joo, H.; Mosberg, H. I.; Lomize, A. L., OPM database and PPM web server: resources for positioning of proteins in membranes. *Nucleic Acids Res.* **2011,** 40, (D1), D370-D376.
13. Gupta, R.; Jung, E.; Brunak, S., Prediction of N-glycosylation sites in human proteins. **2004,** 46, 203-206.
14. Taherzadeh, G.; Dehzangi, A.; Golchin, M.; Zhou, Y.; Campbell, M. P., SPRINT-Gly: predicting N- and O-linked glycosylation sites of human and mouse proteins by using sequence and predicted structural properties. *Bioinformatics* **2019,** 35, (20), 4140-4146.
15. Thomas, S., The Structure of the Membrane Protein of SARS-CoV-2 Resembles the Sugar Transporter SemiSWEET. *Pathogens & immunity* **2020,** 5, (1), 342-363.
16. Yang, J.; Yan, R.; Roy, A.; Xu, D.; Poisson, J.; Zhang, Y., The I-TASSER Suite: protein structure and function prediction. *Nature Methods* **2015,** 12, (1), 7-8.
17. Abraham, M. J.; Murtola, T.; Schulz, R.; Páll, S.; Smith, J. C.; Hess, B.; Lindahl, E., GROMACS: High performance molecular simulations through multi-level parallelism from laptops to supercomputers. *SoftwareX* **2015,** 1, 19-25.
18. Lee, J.; Cheng, X.; Swails, J. M.; Yeom, M. S.; Eastman, P. K.; Lemkul, J. A.; Wei, S.; Buckner, J.; Jeong, J. C.; Qi, Y., CHARMM-GUI input generator for NAMD, GROMACS, AMBER, OpenMM, and CHARMM/OpenMM simulations using the CHARMM36 additive force field. *Journal of chemical theory and computation* **2016,** 12, (1), 405-413.
19. Park, S.-J.; Lee, J.; Qi, Y.; Kern, N. R.; Lee, H. S.; Jo, S.; Joung, I.; Joo, K.; Lee, J.; Im, W., CHARMM-GUI Glycan Modeler for modeling and simulation of carbohydrates and glycoconjugates. *Glycobiology* **2019,** 29, (4), 320-331.
20. Lan, J.; Ge, J.; Yu, J.; Shan, S.; Zhou, H.; Fan, S.; Zhang, Q.; Shi, X.; Wang, Q.; Zhang, L.; Wang, X., Structure of the SARS-CoV-2 spike receptor-binding domain bound to the ACE2 receptor. *Nature* **2020,** 581, (7807), 215-220.
21. Walls, A. C.; Park, Y.-J.; Tortorici, M. A.; Wall, A.; McGuire, A. T.; Veesler, D., Structure, function, and antigenicity of the SARS-CoV-2 spike glycoprotein. *Cell* **2020,** 181, (2), 281-292. e6.
22. Verba, K.; Gupta, M.; Azumaya, C.; Moritz, M.; Pourmal, S.; Diallo, A.; Merz, G.; Jang, G.; Bouhaddou, M.; Fossati, A.; Brilot, A.; Diwanji, D.; Hernandez, E.; Herrera, N.; Kratochvil, H.; Lam, V.; Li, F.; Li, Y.; Nguyen, H.; Nowotny, C.; Owens, T.; Peters, J.; Rizo, A.; Schulze-Gahmen, U.; Smith, A.; Young, I.; Yu, Z.; Asarnow, D.; Billesbølle, C.; Campbell, M.; Chen, J.; Chen, K. H.; Chio, U. S.; Dickinson, M.; Doan, L.; Jin, M.; Kim, K.; Li, J.; Li, Y. L.; Linossi, E.; Liu, Y.; Lo, M.; Lopez, J.; Lopez, K.; Mancino, A.; Iii, F. M.; Paul, M.; Pawar, K.; Pelin, A.; Pospiech, T.; Puchades, C.; Remesh, S.; Safari, M.; Schaefer, K.; Sun, M.; Tabios, M.; Thwin, A.; Titus, E.; Trenker, R.; Tse, E.; Tsui, T. K. M.; Feng, F.; Zhang, K.; Zhang, Y.; Zhao, J.; Zhou, F.; Zhou, Y.; Zuliani-Alvarez, L.; Agard, D.; Cheng, Y.; Fraser, J.; Jura, N.; Kortemme, T.; Manglik, A.; Southworth, D.; Stroud, R.; Swaney, D.; Krogan, N.; Frost, A.; Rosenberg, O., CryoEM and AI reveal a structure of SARS-CoV-2 Nsp2, a multifunctional protein involved in key host processes. *Res Sq* **2021**.
23. Ma, J.; Chen, Y.; Wu, W.; Chen, Z., Structure and Function of N-Terminal Zinc Finger Domain of SARS-CoV-2 NSP2. *Virologica Sinica* **2021**.
24. Maier, J. A.; Martinez, C.; Kasavajhala, K.; Wickstrom, L.; Hauser, K. E.; Simmerling, C., ff14SB: Improving the Accuracy of Protein Side Chain and Backbone Parameters from ff99SB. *Journal of Chemical Theory and Computation* **2015,** 11, (8), 3696-3713.
