# Supplementary material for: Dissecting Phenotype from Genotype with Clinical Isolates of SARS-CoV-2 First Wave Variants
Source: Viruses. 2023 Feb 23;15(3):611. doi: 10.3390/v15030611 (PMC10059853; doi:10.3390/v15030611)
Supplement: Supplementary file 1 [file viruses-15-00611-s001.zip › Supplementary B/Table S1_Amplicon Primers.pdf]

TABLE S1. UT-V1 primers used for disjointed amplicon tiling approach

Primer No.	Primer Pool	Forward Primer Genome Location	Sequence	Reverse Primer Genome Location	Sequence
1	2	1-28	WTKRWWGGTTTATACCTTCCCAGGTAAC	533-556	GTACTGAATGCCTTCGAGTTCTGC
2	3			1055-1077	GGACATTCCCCATTGAAGGTGTC
3	1	472-494	GTTCATCAAACGTTCGGAWGCTC	1544-1568	GGTTACAACCTATGTTAGCGYTAGC
4	2	1006-1031	GAGCTATGAATTGCAGACACCTTTTG	2142-2164	CTCTTCAAGCCAATCAAGGACGG
5	3	1485-1509	GAGGCTGTGTGTTCTCTTATGTTGG	2567-2592	GCTTCAACAGCTTCACTAGTAGGTTG
6	1	2061-2086	CCTACATTACAGGTGGTGTTGTTCAG	3044-3068	CACCTTCTTCTTCATCCTCATCTGG
7	2	2494-2516	GGGAGAAACACTTCCCACAGAAG	3539-3564	CCCACTTTAAGTGGTCCATTAGTAGC
8	3	2993-3018	GAGTCTGGTGAGTTTAAATTGGCTTC	4139-4163	CCACAGCAGTTAAAACACCCTCTTG
9	1	3473-3496	GGTGTTGCAGGAGCCTTAAATAAG	4556-4583	CGTTAAGTGTGTTGATAAGTGACGCTAC
10	2	4067-4093	GCCACTCTTGTTAGTGACATTGACATC	5031-5055	GTTGGACCAAACTGTTGTCCATATG
11	3	4499-4523	CAAGAGGGTGTGGTTGATTATGGTG	5609-5633	CACACGTACAAGGTATCTGAACACC
12	1	4983-5008	CAGTAGACAACATTAACCTCCACACG	5988-6012	GGTACAAGATCAATTGGTTGCTCTG
13	2	5532-5555	GACAACAGCAGACAACCCTTAAGG	6530-6553	CATTAGATCTGTGTGGCCAACCTC
14	3	5921-5946	GGTGTTGTTTGTACAGAAATTGACCC	7042-7067	CTCTGTAACCAGTACAGTAAGAAGGC
15	1	6428-6454	CCTACCATACAGAAAGACGTTCTTGAG	7515-7539	GTTGTACATTCGACTCTTGTTGCTC
16	2	6997-7019	CTACTCAACCGCTGCTTTAGGTG	8003-8025	GCAACTTCCGCACTATCACCAAC
17	3	7457-7485	GTGCATGTTGTAGACGGTTGTAATTCATC	8488-8513	CTTGTCTAGTAGTTGCACATGTCAAC
18	1	7932-7955	CAGCGTCTGTTTACTACAGTCAGC	9035-9057	GGTACTGGCTTACCAGAAGCATC
19	2	8382-8408	GTCACAACATTGCTTTGATATGGAACG	9422-9447	GCAAGGCATGTTACTACGATAGCTAC
20	3	8975-9000	GAGTACACTGACTTTGCAACATCAGC	10073-10097	CCTCAACTTTACCAGATGGGAATGC
21	1	9363-9389	CACTAATTCAACCTATTGGTGCTTTGG	10517-10543	GTGCATGTAACAAAAAGAGACACAGTC
22	2	9998-10023	GGTTCTGATGTTCTTTACCAACCACC	10958-10981	CACTGCACTTTGGAAAGTAACACC
23	3	10432-10456	CCAATGTGCTATGAGGCCCAATTTC	11516-11540	CAATACCTCTGGCCAAAAACATGAC
24	1	10886-10908	GGACGTACCATATTGGGTAGTGC	12137-12161	GCTCATAAGCTTCTTGAGCAGTAGC
25	2	11441-11466	GCTTTAGATCAAGCCATTTCCATGTG	12613-12638	GCCATGCTAAATTAGGTGAATTGTCC
26	3	12073-12098	CAACAGGGCAACCTTACAAGCTATAG	13072-13094	CAGCATCTACAGCAAAAGCACAG
27	1	12553-12582	GGAAATCCAACAGGTTGTAGATGCAGATAG	13511-13536	GTATACGACATCAGTACTAGTGCCTG
28	2	12981-13007	GAGGTATGGTACTTGGTAGTTTAGCTG	14181-14202	CTCTGCAGTTAAAGCCCTGGTC
29	3	13459-13484	GTTTTTAAACGGGTTTGCGGTGTAAG	14547-14572	GGTCAGCAGCATACACAAGTAATTCC
30	1	14106-14128	CATACAAACCACGCCAGGTAGTG	15088-15109	CGGTGCGAGCTCTATTCTTTGC

31	2	14478-14503	CCACTTCAGAGAGCTAGGTGTTGTAC	15540-15566	GATAAAAGTGCATTAACATTGGCCGTG
32	3	14913-14937	CCAAGTCATCGTCAACAACCTAGAC	16005-16028	GCTAAAGACACGAACCGTTCAATC
33	1	15485-15508	CAGGAGATGCCACAACTGCTTATG	16526-16551	GTCAGTAACATTATCGCTACCAACAC
34	2	15921-15944	GTACCTTCCTTACCCAGATCCATC	17134-17158	CTGTATACACTATGCGAGCAGAAGG
35	3	16475-16501	GTTTTCCATTGTGTGCTAATGGACAAG	17528-17552	CCGAGGAACATGTCTGGACCTATAG
36	1	17064-17087	GTATTCTACACTCCAGGGACCACC	18086-18109	GTGCCTGTGTAGGATGTAACCCAG
37	2	17462-17485	GCACATTGCTAACTAAGGGCACAC	18508-18534	CTTTATACGCACTACATTCCAAGGAAG
38	3	18013-18037	CCACGTAGGAATGTGGCAACTTTAC	19089-19113	GTCACTACAAGGCTGTGCATCATAG
39	1	18457-18481	CCACCGCCTGGAGATCAATTTAAAC	19542-19565	CACAAGCTAAAGCCAGCTGAGATC
40	2	19018-19042	CCAGTTCTTCACGACATTGGTAACC	19990-20016	GTCTACTTGACCATCAACTCTACCATC
41	3	19478-19501	GTGGTGCTGTCTGTAGACATCATG	20610-20635	CTACATGGCCATCTTTACACCAAAGC
42	1	19932-19957	GACTGACATAGCCAAGAAACCAACTG	20962-20987	GTTGAATCTGCATCAGAGACAAAGTC
43	2	20469-20493	GCAAACAGGTTCATCTAAGTGTGTG	21679-21704	CTGAGGATCTGAAAACTTTGTCAGGG
44	3	20905-20927	GCTGTTTTAAGACAGTGGTTGCC	21896-21917	GTAGGGACTGGGTCTTCGAATC
45	1	21632-21657	CAATTACCCCCTGCATACACTAATTC	22630-22653	GCAACACAGTTGCTGATTCTCTTC
46	2	21808-21833	CCCTGTCCTACCATTTAATGATGGTG	22976-22998	GTGCTACCGGCCTGATAGATTTC
47	3	22574-22599	CCTTTTGGTGAAGTTTTTAACGCCAC	23627-23652	GTGTAGGCAATGATGGATTGACTAGC
48	1	22856-22878	GGCTGCGTTATAGCTTGGAATTC	24098-24120	GCACAAATGAGGTCTCTAGCAGC
49	2	23570-23596	GGTATATGCGCTAGTTATCAGACTCAG	24512-24536	CTTCAGCCTCAACTTTGTCAAGACG
50	3	24056-24080	GCTGGCTTCATCAAACAATATGGTG	25109-25133	CAACCTCATTGAGGCGGTCAATTTC
51	1	24445-24469	CACGCTTGTTAAACAACTTAGCTCC	25447-25472	GAAGGAGTAGCATCCTTGATTTCACC
52	2	25052-25078	GATGTTGATTTAGGTGACATCTCTGGC	26104-26128	GGACATGTTCTTCAGGCTCATCAAC
53	3	25352-25376	CCAGTGCTCAAAGGAGTCAAATTAC	26453-26478	CGTTTAGACCAGAAGATCAGGAACTC
54	1	26031-26057	CCAGCTGTACTCAACTCAATTGAGTAC	27093-27117	GACTGTATGCAGCAAAACCTGAGTC
55	2	26373-26398	CTGCTGCAATATTGTTAACGTGAGTC	27493-27517	CGTATGTTCCAGAAGAGCAAGGTTC
56	3	27001-27024	GTGACATCAAGGACCTGCCTAAAG	27990-28014	GAATAGGACACGGGTCATCAACTAC
57	1	27428-27453	CACTCGCTACTTGTGAGCTTTATCAC	28534-28559	GAATTCGTCTGGTAGCTCTTCGGTAG
58	2	27931-27955	CTGTAGCTGCATTTCACCAAGAATG	29025-29047	CTTCTTAGAAGCCTCAGCAGCAG
59	3	28481-28504	GGACAAGGCGTTCCAATTAACACC	29551-29576	CGTTTATATAGCCCATCTGCCTTGTG
60	1	28971-28994	GCAAAATGTCTGGTAAAGGCCAAC	29844-29873	KTYWTTYTYYYRAGARGYTMTYMAAATCAC
62	2	29487-29512	CCAAACAATTGCAACAATCCATGAGC		
