# Supplementary material for: Dissecting Phenotype from Genotype with Clinical Isolates of SARS-CoV-2 First Wave Variants
Source: Viruses. 2023 Feb 23;15(3):611. doi: 10.3390/v15030611 (PMC10059853; doi:10.3390/v15030611)
Supplement: Supplementary file 1 [file viruses-15-00611-s001.zip › Supplementary B/Table S2_MinION Sequencing Results.pdf]

TABLE S2. MinION Sequencing Results

Specimen ID No.	Primer Set	Total Length (Gb)	N Count	Percent Genome Coverage	qRT-PCR Ct 1 (ORF1ab gene) [a]	qRT-PCR Ct 2 (E gene) [a]	Lineage (Pangolin)
Lab Reference USA-WA1/2020	UT-V1	1.53	91	100	N/A	N/A	A
UT1	UT-V1	0.1	10472	65	32.26	34.63	ns
UT2	UT-V1	1.25	388	99	24.26	24.84	B.1.324
UT3	UT-V1	0.35	971	97	31.41	34.28	B.1.184
UT4	UT-V1	0.35	5263	82	32.11	35.38	ns
UT5	UT-V1	3.84	95	100	18.29	18.04	B.1.595
UT6	UT-V1	0.41	2826	91	34.82	35.79	ns
UT7	UT-V1	0.15	25886	13	34.31	38.75	ns
UT8	UT-V1	0.76	389	99	25.55	26.08	B.1.110.3
UT9	UT-V1	1.4	384	99	23.09	23.92	B.1.110.3
UT10	UT-V1	3.01	92	100	16.64	16.51	B.1.595
UT11	UT-V1	0.26	406	99	30.94	31.73	B.1.595
UT12	UT-V1	1.27	384	99	24.65	25.04	B.1
UT13	UT-V1	0.46	23089	23	32.76	34.5	ns
UT14	UT-V1	1.84	384	99	25.03	25.95	B.1
UT15	UT-V1	4.75	384	99	17.85	18.28	B.1
UT16	UT-V1	0.69	568	98	30.72	32.81	B.1.567
UT17	UT-V1	0.33	26755	11	32.65	34.84	ns
UT18	UT-V1	0.94	860	97	29.3	30.14	B.1.369
UT19	UT-V1	0.73	1402	95	30.93	32.8	B.1
UT20	UT-V1	0.86	384	99	28.96	29.97	B.1
UT21	UT-V1	1.71	388	99	20.04	20.36	A.1
UT22	ARTIC V3	0.77	121	100	24.93	25.65	B.1
UT23	ARTIC V3	0.6	122	100	23.68	23.85	B.1
UT24	ARTIC V3	0.04	8908	70	31.82	33.22	ns
UT25	ARTIC V3	0.1	8368	72	32.59	34.79	ns
UT26	ARTIC V3	0.12	4661	84	32.06	34.94	ns
UT27	ARTIC V3	0.92	121	100	15.63	15.76	B.1.411
UT28	ARTIC V3	0.88	121	100	17.78	18.15	B.1.411
UT29	ARTIC V3	0.71	121	100	15.83	16	B.1
UT30	ARTIC V3	0.69	121	100	15.07	15.5	B.1.12
UT31	ARTIC V3	0.7	121	100	27.44	28.17	B.1
UT32	ARTIC V3	0.64	124	100	21.28	21.69	B.1
UT33	ARTIC V3	0.58	122	100	25	25.33	B.1.411
UT34	ARTIC V3	0.95	122	100	14.83	15.06	B.1
UT35	ARTIC V3	0.54	1445	95	31.43	32.61	B.1.1.307
UT36	ARTIC V3	2	150	99	21.93	22.09	B.1.1
UT37	ARTIC V3	0.21	4059	86	30.47	32.84	ns
UT38	ARTIC V3	0.51	1763	94	30.42	31.16	B.1.446
UT39	ARTIC V3	1.58	701	98	25.59	25.63	B.1
UT40	ARTIC V3	0.68	1113	96	29.1	30.23	B.1.360
UT41	ARTIC V3	0.42	7528	75	31.41	33.8	ns
UT42	ARTIC V3	0.26	2775	91	30.82	32.3	ns
UT43	ARTIC V3	0.1	28488	5	32.74	38.26	ns
UT44	ARTIC V3	0.28	1022	97	22.07	22.68	B.1.595
UT45	ARTIC V3	1.05	979	97	30.18	30.6	B.1

[a] qRT-PCR Ct values were tested on the Roche Cobas 6800/8000 instrument; Abbreviations: ns – sequence quality was not sufficient for phylogenetic analysis; N count – total number of ‘N’ or unknown bases in the consensus sequence
