# Supplementary material for: Dissecting Phenotype from Genotype with Clinical Isolates of SARS-CoV-2 First Wave Variants
Source: Viruses. 2023 Feb 23;15(3):611. doi: 10.3390/v15030611 (PMC10059853; doi:10.3390/v15030611)
Supplement: Supplementary file 1 [file viruses-15-00611-s001.zip › Supplementary B/Table S4_Model Fit Parameters.pdf]

TABLE S4. Model Fit Parameters

		WA1/2020	UT21	UT29	UT5	UT23
Female	Virus production (p)	2.94 [0.74,19.62]	2.45 [1.68,21.02]	8.20 [2.93,41.41]	2.14 [1.21,13.73]	1.22 [1.05,15.57]
	Virus clearance (c)	8.73 [2.33,50.0]	6.87 [3.74,49.67]	8.41 [3.02,47.58]	18.10 [11.45,49.70]	4.29 [3.38,48.21]
	Infectivity (β) x10^5	1.22 [0.42, 4.66]	1.60 [0.1,2.32]	0.44 [0.12,2.96]	4.70 [1.08,420]	6.45 [0.77,481]
	Infected cell clearance (δ)	1.70 [1.39,1.92]	1.69 [1.37,2.06]	4.51 [2.62,5.35]	1.46 [1.17,1.73]	1.16 [0.93,1.38]
Male	Virus production (p)	0.42 [0.10,12.93]	0.55 [0.10,7.11]	0.95 [0.38,15.41]	1.05 [0.77,4.35]	0.10 [0.01,2.13]
	Virus clearance (c)	10.0 [8.30,50.0]	10.0 [8.30,48.77]	5.33 [4.48,45.75]	10.0 [8.43,49.50]	3.09 [0.06,49.77]
	Infectivity (β) x 10^5	5.0 [0.1,7.72]	4.65 [0.915,6.0]	4.06 [0.66,8.13]	5.0 [2.38,244]	5.0 [0.64,90.4]
	Infected cell clearance (δ)	1.22 [0.98,1.47]	0.97 [0.48,1.16]	1.34 [1.07,1.61]	0.96 [0.77,1.14]	3.07 [2.46,3.66]

Parameter values obtained from fitting Equations 1-4 to viral loads from female and male mice infected with WA1/2020, UT21, UT29, UT5 or UT23. The infected cell clearance for female mice was fixed and allowed to vary within ±20%.
