# Supplementary material for: Dissecting Phenotype from Genotype with Clinical Isolates of SARS-CoV-2 First Wave Variants
Source: Viruses. 2023 Feb 23;15(3):611. doi: 10.3390/v15030611 (PMC10059853; doi:10.3390/v15030611)
Supplement: Supplementary file 1 [file viruses-15-00611-s001.zip › Supplementary B/Table S6_Neutrophil counts.pdf]

TABLE S6. Neutrophil counts at 5 dpi within sections of right lung lobes of infected mice

Treatment	Mouse ID #	Sex	Lung lobe 1 (cells/ m^2)	Lung lobe 2 (cells/ m^2)	Lung lobe 3 (cells/ m^2)	Lung lobe 4 (cells/ m^2)	Average cells/m^2
Mock	F49	F	23	34	N/A	N/A	26
	F263	F	48	32	33	N/A	38
	M299	M	28	36	43	N/A	36
	M7	M	32	40	N/A	N/A	36
	F208	F	17	23	23	N/A	21
	F209	F	25	29	29	N/A	28
WA1/2020	F288	F	18	13	23	N/A	18
	M8	M	13	15	17	21	17
	F216	F	44	49	75	81	62
	M222	M	8	14	13	19	14
	M223	M	32	30	22	13	24
UT5	F228	F	18	19	24	30	23
	F229	F	13	10	11	21	14
	M234	M	16	16	24		19
	M235	M	14	20	23	33	19
UT21	F215	F	58	32	22	44	39
	M11	M	25	29	29	N/A	28
	F57	F	105	63	60	58	72
	M10	M	105	60	51	46	66
UT23	F240	F	25	26	26	35	28
	F241	F	41	38	23	N/A	38
	M246	M	23	22	20	15	20
	M247	M	63	39	61	N/A	54
UT29	F276	F	32	34	24	19	27
	F140	F	26	33	35	40	34
	M9	M	12	10	19	27	16
	M101	M	7	8	16	N/A	10

N/A – lobe not present in tissue sectio
