# Supplementary material for: Dissecting Phenotype from Genotype with Clinical Isolates of SARS-CoV-2 First Wave Variants
Source: Viruses. 2023 Feb 23;15(3):611. doi: 10.3390/v15030611 (PMC10059853; doi:10.3390/v15030611)
Supplement: Supplementary file 1 [file viruses-15-00611-s001.zip › Supplementary B/Table S7_Mouse gene expression profiles.pdf]

TABLE S7. Mouse gene expression

	WA1/2020			UT21			UT29			UT5			UT23		
	D1	D3	D5	D1	D3	D5	D1	D3	D5	D1	D3	D5	D1	D3	D5
ACE2	2.2	2.1	0.7	1.9	1.5	0.9	2.3	1.8	0.3	2.5	2.2	0.8	1.7	2.0	1.1
AGTR2	2.9	2.9	1.3	2.3	2.3	1.8	3.1	2.5	1.7	3.3	2.4	1.3	2.3	3.0	1.3
CCL2	4.7	8.1	4.7	3.6	7.7	2.6	4.2	8.1	4.7	5.7	8.9	5.3	2.8	8.6	3.8
CCL3	0.9	2.8	2.5	0.3	2.4	1.1	0.8	2.3	2.3	1.5	2.9	2.8	0.4	3.2	2.0
CXCL10	6.4	9.7	5.3	5.7	9.3	3.9	6.3	9.4	5.5	7.9	10.0	5.6	5.1	10.4	4.9
DPP4	0.2	0.1	-0.8	-0.4	0.0	-0.7	-0.1	-0.5	-1.2	0.6	0.4	-1.0	0.3	0.4	-0.6
IFNG	3.4	3.9	3.8	3.0	3.4	2.5	3.4	3.7	3.2	3.6	3.9	4.4	2.6	4.1	3.9
IL10	2.4	-0.4	-0.6	2.0	-1.5	-0.8	2.6	-1.3	-0.7	2.9	-0.6	-0.2	3.3	-0.3	0.2
IL6	3.8	5.5	3.0	3.0	5.2	2.1	3.7	5.6	3.3	5.0	5.6	3.3	2.8	5.7	2.2
TMPRSS2	0.0	-0.5	-0.3	-0.4	-0.8	0.2	-0.2	-0.9	-0.6	0.5	0.3	-0.6	0.3	-0.5	0.2
TNF	1.8	2.7	1.6	1.2	2.2	0.6	1.3	2.1	1.7	2.0	2.7	2.1	1.4	3.1	1.3
