# Supplementary figures and images for: Dissecting Phenotype from Genotype with Clinical Isolates of SARS-CoV-2 First Wave Variants
Source: Viruses. 2023 Feb 23;15(3):611. doi: 10.3390/v15030611 (PMC10059853; doi:10.3390/v15030611)

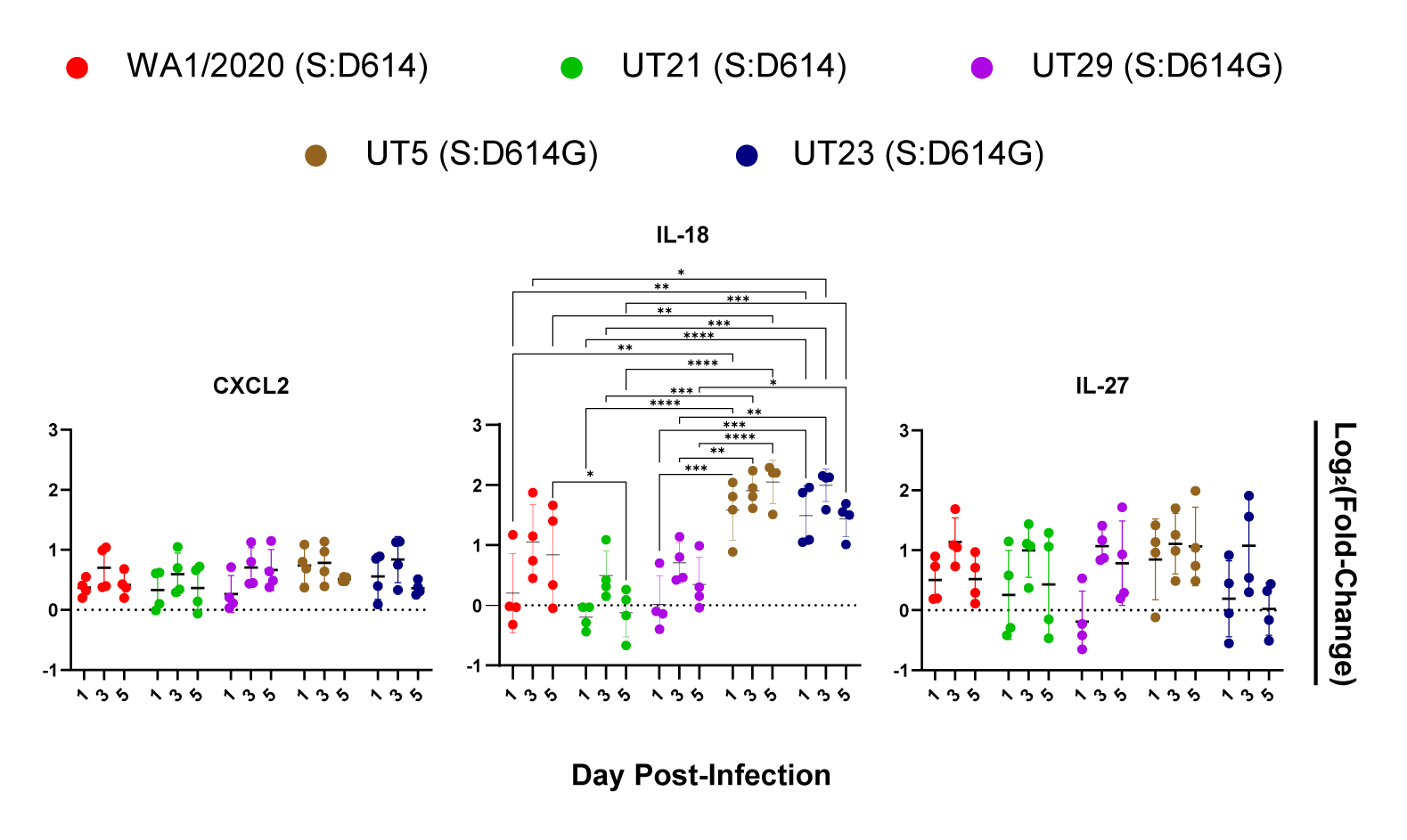

Supplement: Supplementary file 1 [file viruses-15-00611-s001.zip › Supplementary B/Figure S1_Additional_Immune_response.tif]

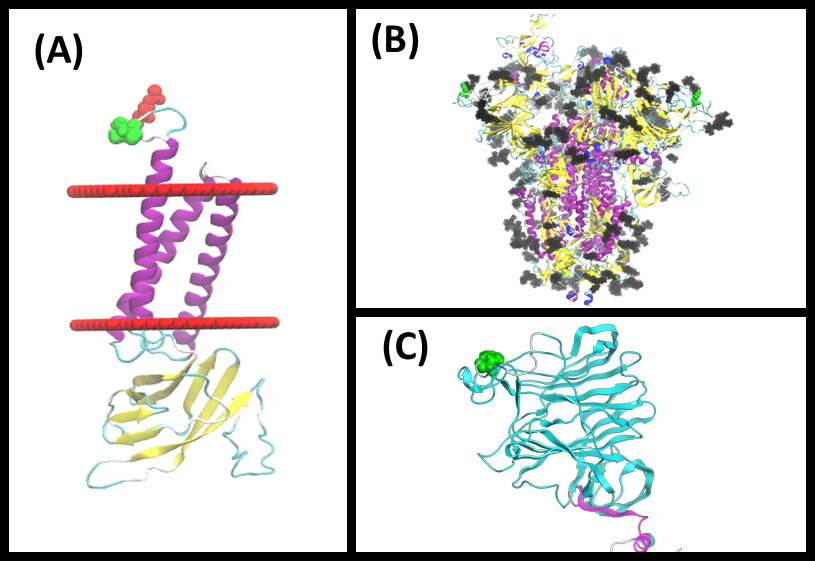

Supplement: Supplementary file 1 [file viruses-15-00611-s001.zip › Supplementary B/Figure S2_renderingImproved.tiff]

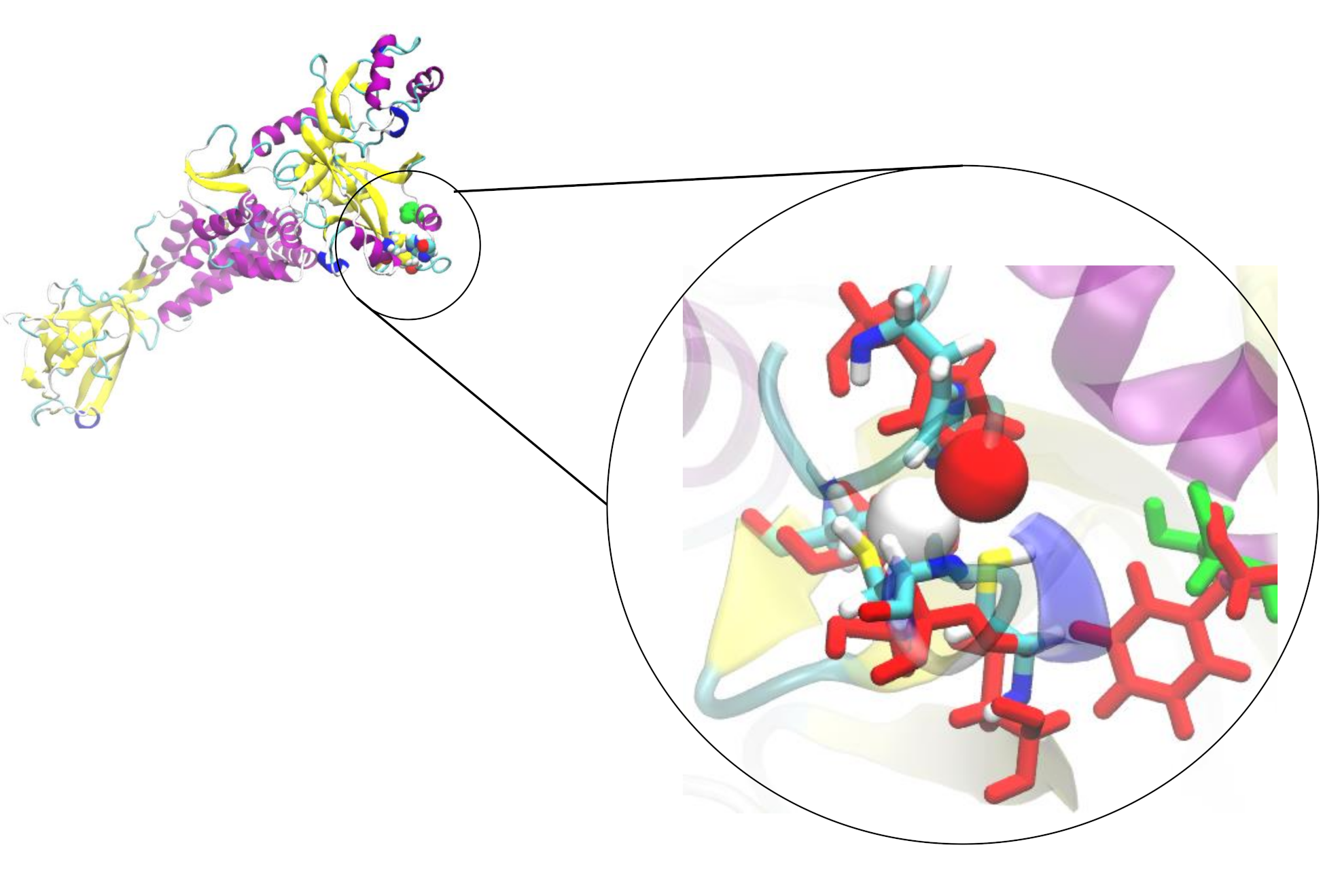

Supplement: Supplementary file 1 [file viruses-15-00611-s001.zip › Supplementary B/Figure S3_nsp2.tiff]
